# Complex probiotics alleviate ampicillin-induced antibiotic-associated diarrhea in mice

**DOI:** 10.3389/fmicb.2023.1156058

**Published:** 2023-04-14

**Authors:** Wenwen Li, Siyu Zhang, Yanyan Wang, Hongsheng Bian, Shuang Yu, Lili Huang, Weiwei Ma

**Affiliations:** College of Pharmacy, Heilongjiang University of Chinese Medicine, Harbin, China

**Keywords:** complex probiotics, antibiotic-associated diarrhea, immunomodulatory, 16S rRNA gene sequencing, gut microbiota

## Abstract

**Aim:**

Antibiotic-associated diarrhea (AAD) is a common side effect during antibiotic treatment, which can cause dysbacteriosis of the gut microbiota. Previous studies have shown beneficial effects in AAD treatment with *Bifidobacterium lactis* XLTG11, *Lactobacillus casei* Zhang, *Lactobacillus plantarum* CCFM8661, and *Lactobacillus rhamnosus* Probio-M9. However, no studies have been conducted on the immunomodulatory effects and protective intestinal barrier function of four complex probiotics. The aim of our study is to investigate the alleviation effects of complex probiotics on ampicillin-induced AAD.

**Methods:**

Thirty-six BALB/c mice were randomly divided into six groups: normal control group (NC), model control group (MC), low-, medium-, and high-dose probiotics groups (LD, MD, and HD), and positive drug (Bifico, 1 × 10^7^ cfu) control group (PDC; Bifico, also known as Bifidobacterium Triple Live Capsule, is composed of *Bifidobacterium longum*, *Lactobacillus acidophilus*, and *Enterococcus faecalis*). An AAD model was established by intragastric administration of ampicillin, by gavage of different doses of complex probiotics and Bifico. The weight gain, fecal water content, loose stool grade, intestinal permeability, total protein and albumin levels, intestinal barrier, cytokine levels, and gut microbiota were determined.

**Results:**

The results showed that complex probiotics significantly decreased the fecal water content, loose stool grade, intestinal permeability, and ileum tissue damage. Their application increased the weight gain, SIgA, TP, and ALB levels. Additionally, complex probiotics significantly decreased the levels of pro-inflammatory cytokines and increased those of anti-inflammatory cytokines. Meanwhile, the mRNA expression levels of ZO-1, occludin, claudin-1, and MUC2 were significantly upregulated in the probiotic-treated group. Furthermore, the complex probiotics increased the gut microbiota diversity and modulated the changes in the gut microbiota composition caused by ampicillin. At the phylum level, the abundance of *Proteobacteria* in the HD group was lower than that in the MC group, whereas that of *Bacteroidetes* was higher. At the genus level, the abundances of *Klebsiella* and *Parabacteroides* in the HD group were lower, whereas those of *Bacteroides*, *Muribaculaceae*, and *Lactobacillus* were higher than those in the MC group. Moreover, Spearman’s correlation analysis also found that several specific gut microbiota were significantly correlated with AAD-related indicators.

**Conclusion:**

We found that complex probiotics improved the diarrhea-related indexes, regulated gut microbiota composition and diversity, increased the expression levels of intestinal protective barrier-related genes, preserved the intestinal barrier function, and relieved inflammation and intestinal injury, thereby effectively improving AAD-associated symptoms.

**Graphical Abstract:**
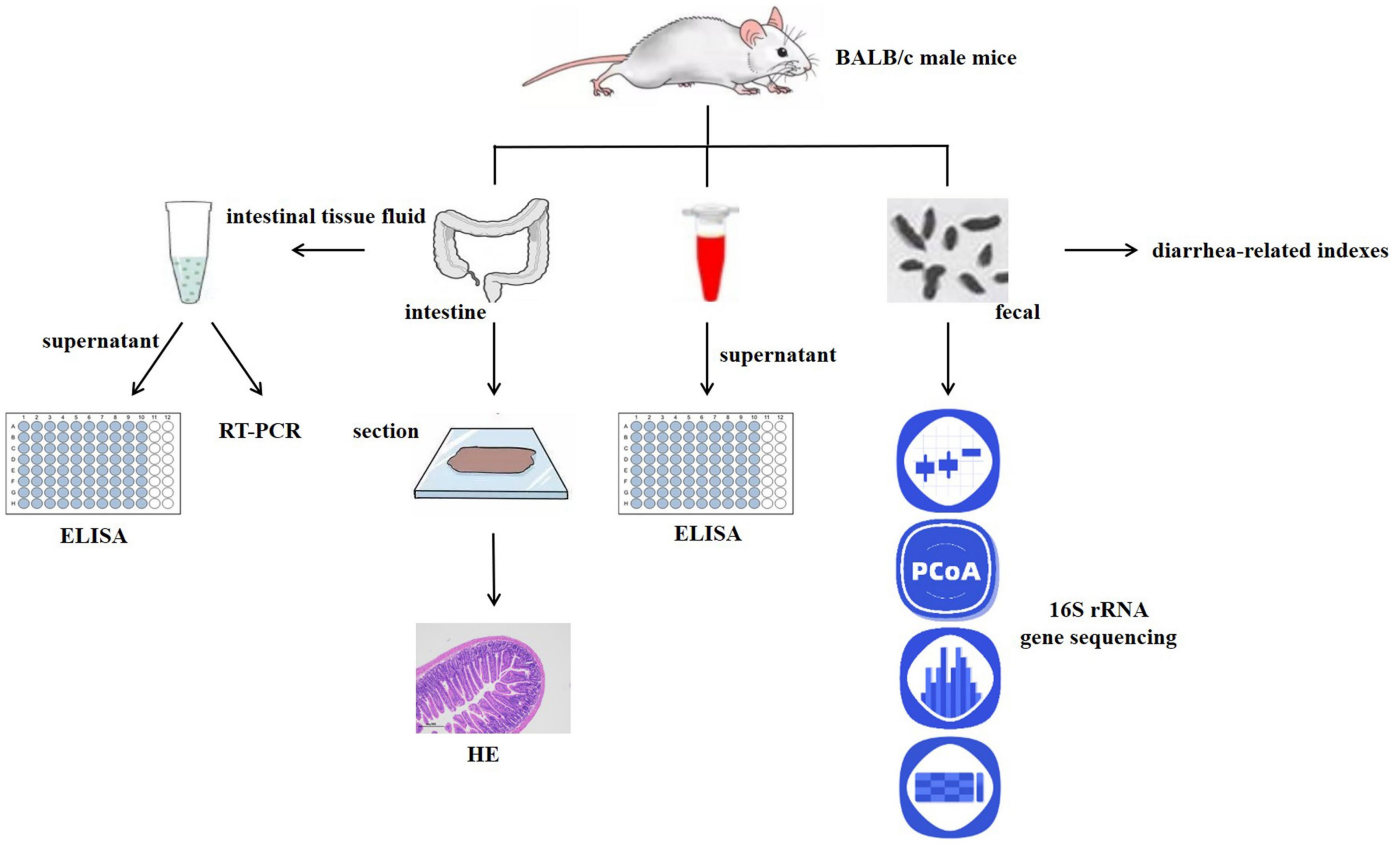

## 1. Introduction

Antibiotics are used to treat illnesses caused by pathogenic infections. Antibiotic-associated diarrhea (AAD) is diarrhea that occurs after the use of antibiotics. It is a common adverse drug reaction (Shao et al., 2020; Yang et al., 2022). Approximately 5–35% of the patients receiving antibiotics have AAD, whose severity ranges from mild to life-threatening (Goodman et al., 2021). The pathogenesis of AAD varies, but the most common mechanism is by damage of the intestinal physiology and microbial community structure caused by antibiotics, resulting in gut dysbiosis (Silverman et al., 2017; Zeng et al., 2019; Mekonnen et al., 2020). Conventional AAD therapy is based on the use of metronidazole and vancomycin and has serious side effects (Silverman et al., 2017). Therefore, it is important to find effective drugs with fewer side effects to alleviate AAD symptoms.

The intestinal tract is the body’s digestive system, and the gut microbiota is the largest and most complex microecological system in the human body. It exerts important immune functions and contributes to host defense against pathogens, plays an important role in the maintenance of intestinal homeostasis, and the development and activation of the host immune system (Nishida et al., 2018; Xu et al., 2023). Probiotics are active microorganisms that are beneficial to the host; they colonize the human body and change the composition of the gut microbiota (Azad et al., 2018; Plaza-Diaz et al., 2019). Probiotics can regulate the gut microbiota environment to improve intestinal health, improve the immune system, and play a beneficial role (Stavropoulou and Bezirtzoglou, 2020; Li Y. et al., 2021). The main immune mechanisms of probiotics include enhancing gastrointestinal mucosal barrier function, activating immune cells, modulating the secretion of immunoglobulins, etc. (La Fata et al., 2018). The supplementation with some probiotics can be done by oral intake; they are safe to use and reportedly had a 7% annual market growth rate worldwide (Zawistowska-Rojek and Tyski, 2018; Peng X. et al., 2022). Probiotics have been confirmed to prevent and improve digestive system diseases, such as acute diarrhea, irritable bowel syndrome (IBS), and AAD (Hempel et al., 2012; Li Z. et al., 2021; Skrzydło-Radomańska et al., 2021; Chen Y. et al., 2022). *Bifidobacteria* and *Lactobacilli* are the most important probiotic bacteria of the gut microbiota (Turroni et al., 2018; Fernández-Ciganda et al., 2022). *Bifidobacteria* was found to exert a protective role in intestinal barrier function in NEC mice by inhibiting proinflammatory cytokine secretion and zonulin protein release, and improving intestinal TJ integrity (Ling et al., 2016). *Bifidobacterium pseudocatenulatum* regulates the gut microbiota activities and immune functions, participates in the intestinal protective barrier, and inhibits the TLR4/NF-κB pathway (Chen et al., 2021). Oral *Lactobacillus* administration increased the Paneth cells counts and the intestinal antimicrobial activity in healthy mice (Cazorla et al., 2018).

Previous research has shown that *Bifidobacterium lactis* XLTG11, *Lactobacillus casei* Zhang, *Lactobacillus plantarum* CCFM8661, and *Lactobacillus rhamnosus* Probio-M9 (Yu et al., 2021; Yao G. et al., 2021; Xu et al., 2022; Zhang et al., 2022) promote immunity and the intestinal protective barrier function. Some studies have shown that probiotic combinations exhibit superior functions in protecting the intestines and regulating the immune system compared to single strains (Chapman et al., 2011). However, the enhancement effects and potential mechanism of complex probiotics consisting of *Bifidobacterium lactis* XLTG11, *Lactobacillus casei* Zhang, *Lactobacillus plantarum* CCFM8661, and *Lactobacillus rhamnosus* Probio-M9 have not been reported. Broad-spectrum penicillins, cephalosporins, and clindamycin have been observed to cause a high occurrence rate of diarrhea. Ampicillin is a broad-spectrum penicillin antibiotic with antibacterial activity against both Gram-positive and Gram-negative bacteria, and can be used for the development of a model of dysbacteriosis diarrhea (Zhang et al., 2019; Xu et al., 2022). The aim of our study was to investigate the alleviation effects of complex probiotics on the gut microbiota of an AAD mouse model. This study will provide a reference for the development of complex probiotics preparations.

## 2. Materials and methods

### 2.1. Experimental materials

#### 2.1.1. Bacterial strain and culture

Complex probiotics powder (*Bifidobacterium lactis* XLTG11, *Lactobacillus casei* Zhang, *Lactobacillus plantarum* CCFM8661, and *Lactobacillus rhamnosus* Probio-M9) was obtained from Jinhua Yinhe Biological Technology Co., Ltd. (Jinhua, China). The complex probiotics powder was stored in a refrigerator at −80°C before the experiment. Normal saline was added to prepare 2.5 × 10^6^, 5 × 10^6^, and 1 × 10^7^ cfu complex probiotics solutions for immediate use.

#### 2.1.2. Animals

Six-week-old specific pathogen-free (SPF) BALB/c male mice were purchased from Liaoning Changsheng Biotechnology Co., Ltd. (Shenyang, China). The animals were raised in an animal room with a barrier environment, kept in a room under controlled temperature (20–23°C) and humidity (30–60%) under a 12:12-h light–dark cycle, with free access to feed and water. All animal procedures were carried out in compliance with Heilongjiang University of Traditional Chinese Medicine’s Regulations on the Administration of Laboratory Animals, and the university’s Animal Ethics Committee approved all experiments (ethic approval code: 2021121201).

### 2.2. Experimental methods

#### 2.2.1. Experimental design

After 1 week of acclimatization, BALB/c mice were randomly divided into six groups (*n* = 6), including the normal control group (NC), model control group (MC), positive drug control group (PDC), low-, medium-, and high-dose complex probiotics groups (LD, MD, and HD). The mice in the MC, PDC, LD, MD, and HD groups (except for those in the NC group) received ampicillin by gavage twice daily at a dose of 11.2 g·kg^−1^ each time. The mice in the NC group were given the same volume of normal saline at the same time every day for 3 consecutive days. After modeling, the mice in the PDC group were given Bifico (1 × 10^7^ cfu) by gavage, and those in the LD, MD, and HD groups were given complex probiotics (2.5 × 10^6^, 5 × 10^6^, and 1 × 10^7^ cfu). For 14 days, the mice in the NC and the MC groups received the same amount of normal saline. After the experiment, blood was sampled from the eyes and stored at −80°C. Fresh feces were collected under sterile conditions and stored at −80°C for gut microbiota analysis. Then, ileum tissue was collected for histopathological analysis and colon tissue for real-time quantitative polymerase chain reaction and ELISA assays.

#### 2.2.2. Weight gain, degree of loose stool, and fecal water content

At 8:00 a.m. on the first day of diarrhea model development, the body weight of the mice in each group was measured and recorded, and then the mice were given ampicillin. At 18:00 every day, the body weight of all mice was also measured and recorded. On the 3rd and 14th day of the experiment, fresh feces of mice in each group were weighed and dried and then weighed again to obtain their dry weight and wet weight, and the degree of loose stool was established using predefined criteria (Table 1).


$$\mathrm{Fecal water content}\;\left(\%\right)=\frac{\mathrm{wet}\;\mathrm{fecal weight}-\mathrm{dry}\;\mathrm{fecal weight}}{\mathrm{wet}\;\mathrm{fecal weight}}\times 100\%$$

**Table 1:** Criteria to determine the degree of loose stool.

Filter paper surface stain diameter/cm	Degree of loose stool
<1	1
1–1.9	2
2–2.9	3
3–3.9	4
4–4.9	5

#### 2.2.3. The levels of total protein and albumin in the serum

Further, 1 h after the last administration, blood was sampled from the eyes and let stand for about 1 h, 3,000 r/min^−1^ centrifuged for 10 min, and then separated the serum. The levels of TP and ALB in the serum samples were determined by ELISA kits (Jiangsu Meimian industrial Co., Ltd., Jiangsu, China).

#### 2.2.4. Assays of intestinal permeability in the serum

The levels of LPS and D-LA in the serum samples were determined by ELISA kits (Jiangsu Meimian industrial Co., Ltd., Jiangsu, China).

#### 2.2.5. Histopathological analysis of ileum

Ileum tissue was fixed in 4% paraformaldehyde for 48 h and then embedded in paraffin (Wuhan Junjie Electronics Co., Ltd., Wuhan, China), and cut into 4-μm sections (Leica Instrument Shanghai Ltd., Shanghai, China). These slices were dewaxed and then stained with hematoxylin–eosin (HE; Servicebio Co., Ltd., Wuhan, China), then photographed and observed under a light microscope (Nikon Eclipse Ci-L, 100× magnification; Nikon, Tokyo, Japan).

#### 2.2.6. Real-time quantitative PCR

Real-time quantitative PCR (RT-PCR) was used to determine the relative mRNA levels of tight junction protein genes (ZO-1, claudin-1, and occludin) and MUC2. Appropriate samples of colon tissue were taken and homogenized. Using the TaKaRa Kit, total RNA was extracted from the colon tissue. Then, mRNA was subjected to reverse transcription into cDNA with PrimeScriptTmRT reagent kit with gDNA Eraser manufactured (TaKaRa). The TB Green® Premix Ex Taq™II kit (TaKaRa) was used to prepare the reaction solution and amplification was performed using a real-time fluorescent quantitative PCR system (QuantStudio 3). The sequences of the primers are presented in Table 1. The following reaction conditions were applied: pre-denaturation 95°C (30 s), PCR 95°C (5 s), 60°C (34 s), repeated for 40 cycles to determine the Ct value of specific gene and the Ct value of β-actin. The expression of genes was examined using the 2^-ΔΔCt^ method (Table 2).

**Table 2:** Design of RT-PCR primer sequences.

Genes	Forward primer (5′-3′)	Reverse primer (5′-3′)
ZO-1	GCGAACAGAAGGAGCGAGAAGAG	GCTTTGCGGGCTGACTGGAG
Occludin	TGGCTATGGAGGCGGCTATGG	AAGGAAGCGATGAAGCAGAAGGC
Claudin-1	GCTGGGTTTCATCCTGGCTTCTC	CCTGAGCGGTCACGATGTTGTC
MUC2	TGCTGACGAGTGGTTGGTGAATG	TGATGAGGTGGCAGACAGGAGAC
β-actin	GGTTGTCTCCTGCGACTTCA	TGGTCCAGGGTTTCTTACTCC

#### 2.2.7. ELISA measurement of inflammatory cytokines of colon

Colon tissues (100 mg) were homogenized in 900 μL of ice-cold PBS using a homogenizer, and the supernatant was then transferred into sterile tubes after centrifugation at 10,000 rpm for 10 min at 4°C. The levels of SIgA, IL-6, IL-1β, and TNF-α in the colon tissue were determined with ELISA kits (Jiangsu Meimian industrial Co., Ltd., Jiangsu, China).

#### 2.2.8. 16S rRNA gene sequencing

The 16S amplicon sequencing and analysis were conducted by OE Biotech Co., Ltd. (Shanghai, China). Next, we utilized the MagPure Soil DNA LQ Kit (Shanghai Magen Biotechnology Co., Ltd., Shanghai, China) to extract genomic DNA from the fecal samples of each group (*n* = 6). The purity and quantity of DNA were confirmed using agarose gel and NanoDrop. Afterward, the bacterial 16S rRNA gene V3-V4 region was amplified using the method/manual of the manufacturer by polymerase chain reaction (PCR) with the forward primer 343F (5′- TACGGRAGGCAGCAG −3′) and the reverse primer 798R (5′- AGGGTATCTAATCCT-3′). Amplicon quality was visualized using gel electrophoresis, purified with Agencourt AMPure Beads (Beckman Coulter, Indianapolis, IN), and amplified for another round of PCR. The final amplicon was measured using the dsDNA HS Assay Kit for Qubit after being once more purified with AMPure XP beads (Yeasen Biotechnology Co., Ltd., Shanghai, China). Sequencing was performed on the Illumina Miseq platform (Illumina Inc., San Diego, CA, United States).

#### 2.2.9. Statistical analysis

The data of the present study were statistically analyzed using SPSS 26.0 software (IBM, Armonk, NY, United States) and are expressed as mean ± SD. One-way ANOVA was employed for comparing differences between groups, followed by Student–Newman–Keuls (S-N-K) test. *p* < 0.05 was considered to indicate a statistically significant difference.

## 3. Results

### 3.1. Effects of complex probiotics on general mice effects in AAD model mice

#### 3.1.1. Effects of complex probiotics on weight gain, fecal water content, and loose stool grade

**Figure 1:**
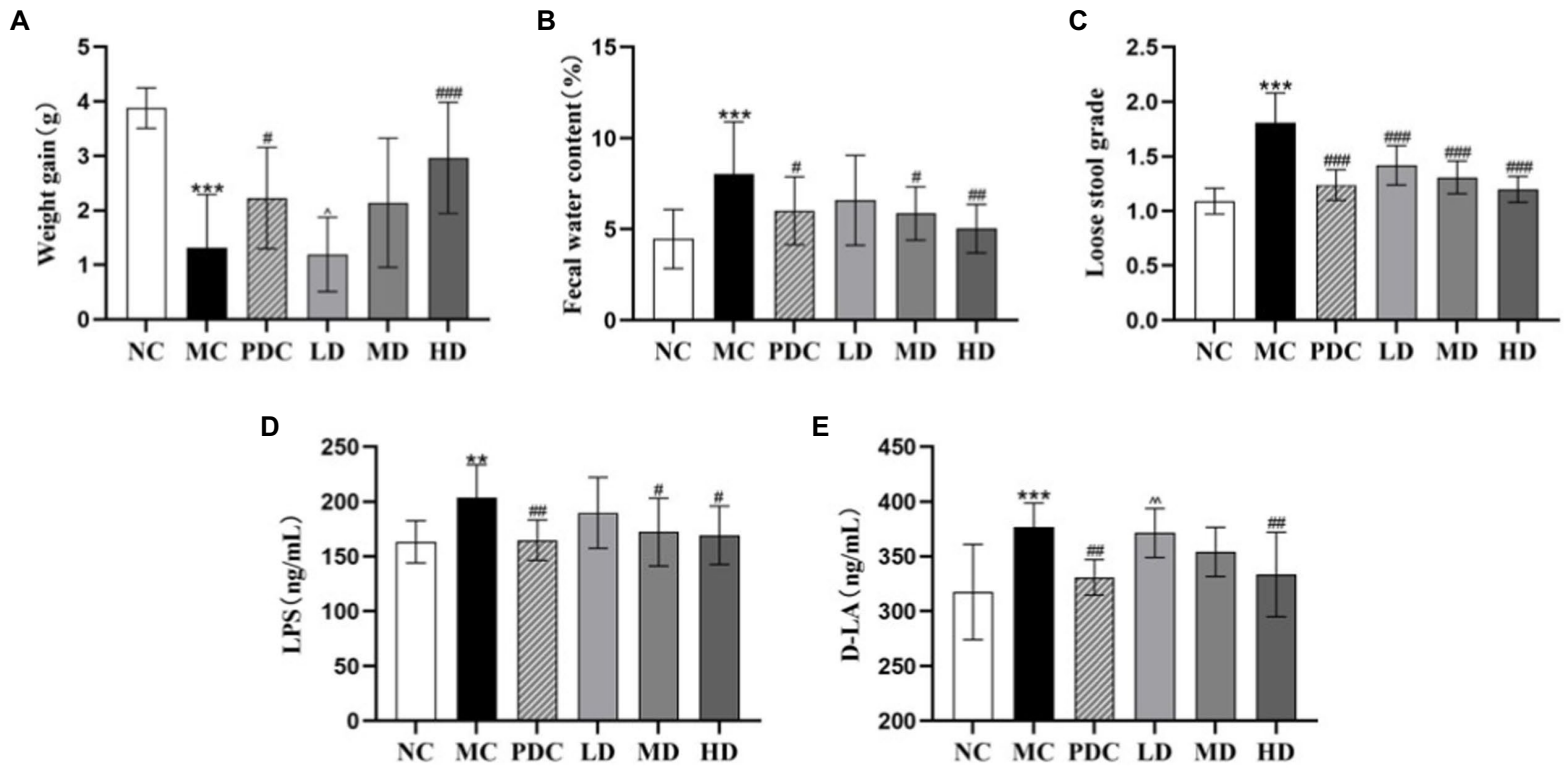
Effects of complex probiotics on general mice effects in AAD model mice (*n* = 6). **(A)** Weight gain; **(B)** Fecal water content; **(C)** Loose stool grade; **(D)** LPS; **(E)** D-LA. NC, normal control group; MC, model control group; PDC, Positive drug control group; LD, low-dose complex probiotics group; MD, medium-dose complex probiotics group; HD, high-dose complex probiotics group. All data are expressed as mean ± SD. Compared with the NC group ^**^p < 0.01, ^***^p < 0.001; Compared with the MC group ^#^*p* < 0.05, ^##^*p* < 0.01, ^###^*p* < 0.001; Compared with the PDC group ^^^*p* < 0.05, ^^^^*p* < 0.01.

Significant differences in the weight gain, fecal water content, and loose stool grade were observed between the NC group and the MC group (*p* < 0.001). Compared with the MC group, the fecal water content of mice in the PDC, LD, MD, and HD groups decreased in a dose-dependent manner by 25.40% (*p* < 0.05), 18.15% (*p* > 0.05), 27.16% (*p* < 0.05), 37.52% (*p* < 0.01), respectively; the loose stool grade decreased by 31.49% (*p* < 0.001), 21.55% (*p* < 0.001), 27.62% (*p* < 0.001), and 33.70% (*p* < 0.001), respectively. Compared with the MC group, the weight gain of the PDC, MD, and HD groups increased by 69.46% (*p* < 0.05), 62.83% (*p* > 0.05), and 125.67% (*p* < 0.001). The weight gain in the LD group was by 46.61% lower (*p* < 0.05) than that in the PDC group. The results showed that the high-dose of the complex probiotics not only significantly improved the diarrhea symptoms of the AAD model mice, but also promoted their growth. The therapeutic effect of Bifico was better than that of the low-dose complex probiotic (Figures 1A–C).

#### 3.1.2. Effects of complex probiotics on intestinal permeability

The contents of LPS and D-LA in the MC group were higher than those in the NC group by 24.87 and 18.59% (*p* < 0.01 or *p* < 0.001), respectively, indicating that ampicillin changed the intestinal permeability of the mice. The content of LPS in the PDC, MD, and HD groups was lower than that in the MC group by 19.15% (*p* < 0.01), 15.46% (*p* < 0.05), and 16.88% (*p* < 0.05), correspondingly; the contents of D-LA in these three groups were lower by 12.14% (*p* < 0.01), 5.99% (*p >* 0.05), and 11.44% (*p* < 0.01), respectively, than that in the MC group. Compared with the PDC group, the content of D-LA in the LD group increased by 12.26% (*p* < 0.01). These findings revealed that the high-dose of complex probiotic had a good effect on reducing intestinal barrier permeability. The therapeutic effect of the low dose of the complex probiotic was weaker than that of Bifico (Figures 1D,E).

### 3.2. Effects of complex probiotics on the index of intestinal immune barrier in AAD model mice

#### 3.2.1. Effects of complex probiotics on serum total protein and albumin

**Figure 2:**
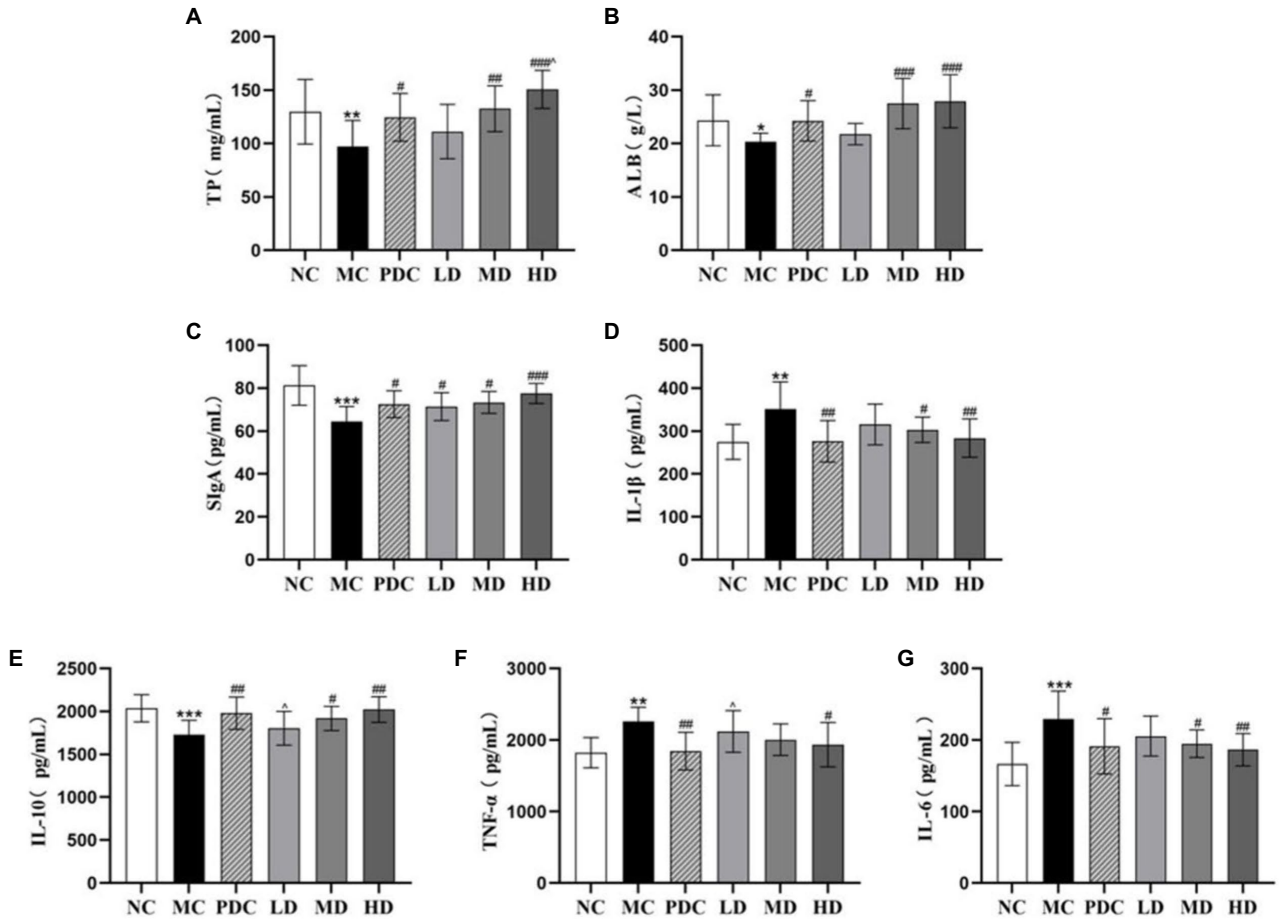
Effects of complex probiotics on the index of intestinal immune barrier in AAD model mice (*n* = 6). **(A)** TP; **(B)** ALB; **(C)** SIgA; **(D)** IL-1β; **(E)** IL-10; **(F)** TNF-α; **(G)** IL-6. NC, normal control group; MC, model control group; PDC, Positive drug control group; LD, low-dose complex probiotics group; MD, medium-dose complex probiotics group; HD, high-dose complex probiotics group. All data are expressed as mean ± SD. Compared with the NC group ^*^p < 0.05, ^**^p < 0.01, ^***^p < 0.001; Compared with the MC group ^#^*p* < 0.05, ^##^*p* < 0.01, ^###^*p* < 0.001; Compared with the PDC group ^^^*p* < 0.05.

Compared with the NC group, the levels of TP and ALB in the serum in the MC group were reduced by 25.09% (*p* < 0.01) and 16.45% (*p* < 0.05). Compared with the MC group, the level of TP in the PDC, MD, and HD groups increased by 28.12% (*p* < 0.05), 36.40% (*p* < 0.01), 55.06% (*p* < 0.001), and the level of ALB increased by 19.32% (*p* < 0.05), 35.23% (*p* < 0.001), and 37.28% (*p* < 0.001). The levels of TP in the HD group were higher than those in the PDC group by 21.02% (*p* < 0.05), indicating that the high-dose complex probiotic have a better therapeutic effect than Bifico (Figures 2A,B).

#### 3.2.2. Effects of complex probiotics on inflammatory cytokines in the colon tissue

We found that the levels of IL-6, IL-1β, and TNF-α in the colon tissue in the MC group were higher than those in the NC group by 37.66% (*p* < 0.001), 27.95% (*p* < 0.01), 23.89% (*p* < 0.01), respectively, whereas the levels of IL-10 and SIgA were lower by 15.01% (*p* < 0.001) and 20.58% (*p* < 0.001), correspondingly. These results indicated that ampicillin triggered inflammation by increasing the secretion of pro-inflammatory factors and decreasing the secretion of anti-inflammatory factors. Compared with the MC group, the levels of SIgA in the PDC, LD, MD, and HD groups increased by 12.33% (*p* < 0.05), 10.66% (*p <* 0.05), 13.67% (*p* < 0.05), and 20.15% (*p* < 0.001), respectively. The levels of IL-6 decreased by 16.54% (*p* < 0.05), 10.31% (*p >* 0.05), 14.97% (*p* < 0.05), and 18.59% (*p* < 0.01), correspondingly. The levels of IL-1β decreased by 21.42% (*p* < 0.01), 10.28% (*p >* 0.05), 13.81% (*p* < 0.05), and 19.34% (*p* < 0.01), respectively, whereas the levels of IL-10 increased by 14.39% (*p* < 0.01), 4.19% (*p >* 0.05), 10.90% (*p* < 0.05), and 16.86% (*p* < 0.01), correspondingly. These results suggested that the complex probiotics decreased the levels of IL-6, IL-1β, and increased the levels of IL-10 in the colon tissue in a dose-dependent manner. Compared with the MC group, the levels of TNF-α in the HD group decreased by 14.29% (*p* < 0.05). However, there was no significant difference in the levels of TNF-α between the MD group and the MC group (*p >* 0.05). Compared with the PDC group, the levels of TNF-α in the LD group increased by 14.80% (*p* < 0.05), the levels of IL-10 decreased by 8.92% (*p* < 0.05). These results showed that the high-dose complex probiotics regulated the level of inflammatory factors and promoted the immune function of the AAD model mice. The therapeutic effect of the low-dose complex probiotic was weaker than that of Bifico (Figures 2C–G).

### 3.3. Effects of complex probiotics on the intestinal in AAD model mice

#### 3.3.1. Effects of complex probiotics on ileum histopathological analysis

**Figure 3:**
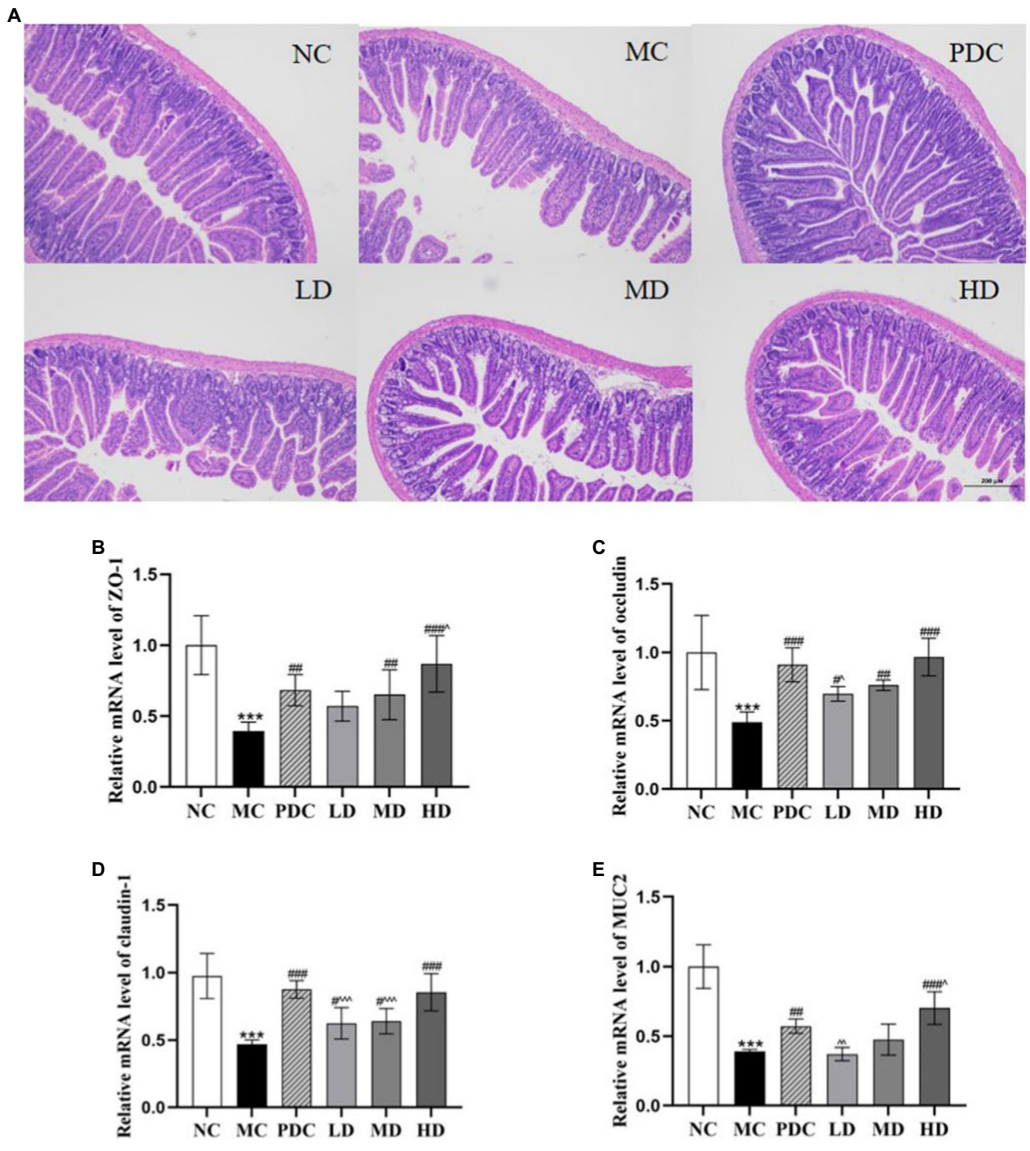
Effects of complex probiotics on the intestinal in AAD model mice (*n* = 6). **(A)** HE staining of jejunum section (×100), **(B)** ZO-1, **(C)** occludin, **(D)** claudin-1, **(E)** MUC2. NC, normal control group; MC, model control group; PDC, Positive drug control group; LD, low-dose complex probiotics group; MD, medium-dose complex probiotics group; HD, high-dose complex probiotics group. All data are expressed as mean ± SD. Compared with the NC group ^***^p < 0.001; Compared with the MC group ^#^*p* < 0.05, ^##^*p* < 0.01, ^###^*p* < 0.001; Compared with the PDC group ^^^*p* < 0.05, ^^^^*p* < 0.01, ^^^^^*p* < 0.001.

The photomicrographs of (HE)-stained intestinal sections are displayed in Figure 3A. In the NC group, the small intestinal mucosa structure was intact, and the villi were arranged closely and neatly. In the MC group, the small intestinal mucosa was damaged severely, and the villi were arranged sparsely. The probiotic administration attenuated the damage degree of the small intestinal mucosa in different degrees in a dose-dependent manner. These findings showed that ampicillin can damage the intestinal structural integrity, whereas the complex probiotic effectively prevented and ameliorated the intestinal damage in a dose-dependent fashion.

#### 3.3.2. Effects of complex probiotics on ZO-1, occludin, claudin-1, and MUC2 mRNA expression in the colon tissue

The mRNA expression levels of ZO-1, occludin, claudin-1, and MUC2 in the MC group were lower than those in the NC group by 60.74% (*p* < 0.001), 51.20% (*p* < 0.001), 51.83% (*p* < 0.001), and 61.24% (*p* < 0.001), respectively, indicating that the AAD model was successfully established. Compared with the MC group, the mRNA expression levels of ZO-1 in the PDC, LD, MD, and HD groups increased by 72.17% (*p* < 0.01), 44.26% (*p* > 0.05), 64.58% (*p* < 0.01), and 119.76% (*p* < 0.001), the mRNA expression levels of occludin increased by 86.40% (*p* < 0.001), 42.83% (*p* < 0.05), 55.74% (*p* < 0.01), and 98.03% (*p* < 0.001), the mRNA expression levels of claudin-1 increased by 86.55% (*p* < 0.001), 33.02% (*p* < 0.05), 36.27% (*p* < 0.05), and 82.05% (*p* < 0.001), and the mRNA expression levels of MUC2 in the PDC and HD groups increased by 47.44% (*p* < 0.01) and 81.03% (*p* < 0.001). However, there was no significant difference in the mRNA expression levels of MUC2 between the MD group and the MC group. Compared with the PDC group, the mRNA expression levels of ZO-1 in the HD group were increased by 27.24% (*p* < 0.05), the mRNA expression levels of occludin and MUC2 in the LD group were reduced by 23.37% (*p* < 0.05) and 35.35% (*p* < 0.01). The mRNA expression levels of MUC2 in the HD group were 22.78% higher (*p* < 0.05), whereas the mRNA expression levels of claudin-1 in the LD and MD groups were lower by 28.69% (*p* < 0.001) and 26.95% (*p* < 0.001), respectively. The aforementioned results indicated that the high dose of the complex probiotics effectively alleviated the damage of the intestinal barrier functional protein in the AAD model mice (Figures 3B–E).

### 3.4. Effects of complex probiotics on the diversity and structure of gut microbiota in AAD model mice

#### 3.4.1. Alpha diversity analysis

**Figure 4:**
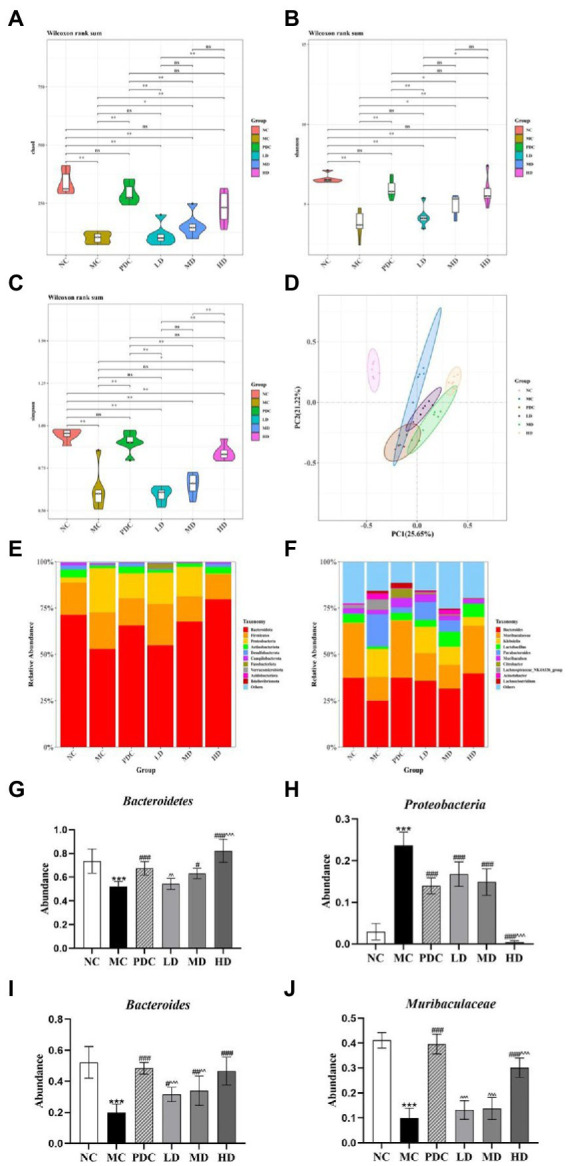
Effects of complex probiotics on the diversity and structure of gut microbiota in AAD model mice (*n* = 6). **(A)** Chao1; **(B)** Shannon; **(C)** Simpson; **(D)** Principle coordinate analysis (PCoA); **(E)** Gut microbiota composition at the phylum level; **(F)** Gut microbiota composition at the genus level; **(G)** The abundance of *Bacteroidetes*; **(H)** The abundance of *Proteobacteria*; **(I)** The abundance of *Bacteroides*; **(J)** The abundance of *Muribaculaceae*. NC, normal control group; MC, model control group; PDC, Positive drug control group; LD, low-dose complex probiotics group; MD, medium-dose complex probiotics group; HD, high-dose complex probiotics group. All data are expressed as mean ± SD. Compared with the NC group ^***^p < 0.001; Compared with the MC group ^#^*p* < 0.05, ^##^*p* < 0.01, ^###^*p* < 0.001; Compared with the PDC group ^^^^*p* < 0.01, ^^^^^*p* < 0.001.

The species richness and evenness of distribution within a sample were assessed by alpha-diversity analysis. The Chao1, Shannon, and Simpson indices were employed for the evaluations in this experiment. The Chao1, Shannon, and Simpson indices in the MC group were significantly lower than those in the NC group (*p* < 0.01). Notably, the administration of the high-dose complex probiotics significantly increased the values of the Chao1, Shannon, and Simpson indices (*p* < 0.05 or *p* < 0.01). The combined use of the complex probiotics effectively promoted the diversity and uniformity of the gut microbiota (Figures 4A–C).

#### 3.4.2. Beta diversity analysis

Principal coordinates analysis was applied to assess community similarity and difference (PCoA). The results showed that the gut microbiota of the MC and NC groups were significantly different, indicating that the AAD model was successfully developed. Significant differences were found between the microflora structure of the MC group and the complex probiotics groups (Figure 4D).

#### 3.4.3. Effects of complex probiotics on the gut microbiota composition in the AAD model mice

At the phylum level, the composition of the intestinal microorganisms in each group at the phylum level included *Bacteroidetes*, *Firmicutes*, *Proteobacteria*, *Epsilonbacteraeota*, and *Actinobacteriota*. Of them, *Bacteroidetes*, *Firmicutes*, and *Proteobacteria* were the dominant flora representatives in the intestinal tract. The relative abundance of *Bacteroidetes* in the MC group (*p* < 0.001) was lower, whereas the relative abundance of *Proteobacteria* was higher (*p* < 0.001). The obtained results indicated that ampicillin transformed the composition of the gut microbiota in the various groups, leading to the overgrowth of *Proteobacteria* but restricted growth of *Bacteroidetes*, which was generally considered to be a feature of dysbiosis. Compared with the MC group, the relative abundance of *Bacteroidetes* in the MD and HD groups increased by 20.93% (*p* < 0.05), 57.76% (*p* < 0.001), and the relative abundance of *Proteobacteria* decreased by 37.06% (*p* < 0.001) and 98.25% (*p* < 0.001). Compared with the PDC group, the relative abundance of *Bacteroidetes* in the LD group decreased by 19.46% (*p* < 0.01), the relative abundance of *Bacteroidetes* in the HD group increased by 21.96% (*p* < 0.001), and the relative abundance of *Proteobacteria* in the HD group decreased by 97.03% (*p* < 0.001). Therefore, the complex probiotics were found to significantly alter the relative abundance of various phyla after antibiotic administration (Figures 4E,G,H).

At the genus level, the composition of the intestinal microorganisms in each group at the genus level included *Bacteroides*, *Muribaculaceae*, *Klebsiella*, *Lactobacillus*, and *Parabacteroides*. Compared with the NC group, the relative abundance of *Bacteroides* and *Muribaculaceae* in the MC group decreased by 62.06% (*p* < 0.001) and 75.78% (*p* < 0.001). The relative abundance of *Klebsiella* and *Parabacteroides* in the MC group were increased. Compared with the MC group, the abundance of *Klebsiella* and *Parabacteroides* in the HD group were reduced, the relative abundance of *Bacteroides* in the MD and HD groups increased by 71.51% (*p* < 0.01), 135.12% (*p* < 0.001), and the relative abundance of *Muribaculaceae* increased by 38.69% (*p* > 0.05), 202.28% (*p* < 0.001). Compared with the PDC group, the relative abundance of *Bacteroides* in the LD and MD groups decreased by 34.77% (*p* < 0.001) and 29.83% (*p* < 0.01), the relative abundance of *Muribaculaceae* in the LD, MD, and HD groups decreased by 66.72% (*p* < 0.001), 65.11% (*p* < 0.001), and 23.97% (*p* < 0.001). The complex probiotics supplementation after antibiotic administration significantly altered the relative intestinal microbial abundance at the genus level (Figures 4F,I,J).

### 3.5. Correlation analysis between diarrhea-related indexes, cytokine levels, intestinal protective barrier-related genes expression, and dominant gut microbiota

**Figure 5:**
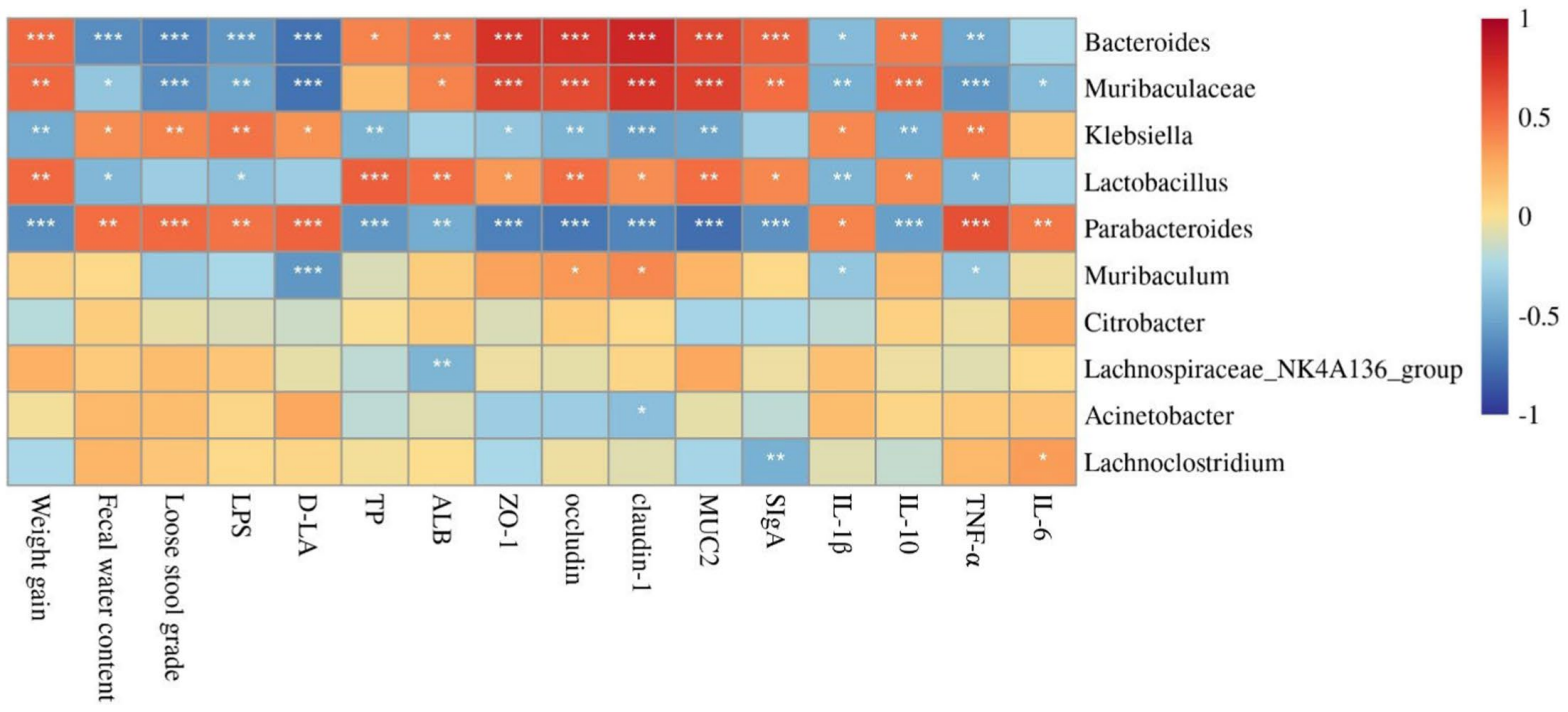
Correlation analysis between diarrhea-related indexes, cytokine levels, intestinal protective barrier-related genes expression, and dominant gut microbiota by Spearman. ^*^, ^**^, and ^***^ indicate the associations significant (*p* < 0.05, *p* < 0.01, and *p* < 0.001, respectively).

To understand the involved and interacted roles of the intestinal microbiota in probiotics-mediated benefits, the correlation analysis between diarrhea-related indexes, cytokine levels, intestinal protective barrier-related genes expression, and dominant gut microbiota at the genus levels was analyzed. The relative abundance of *Bacteroides* and *Muribaculaceae* were significantly positively correlated with weight gain, TP, ALB, tight junction proteins (ZO-1, occludin, and claudin-1), MUC2, SIgA, and IL-10, while was significantly negatively correlated with fecal water content, loose stool grade, LPS, D-LA, and pro-inflammation cytokines (IL-1β, TNF-α, and IL-6). *Klebsiella* and *Parabacteroides* were positively associated with fecal water content, loose stool grade, LPS, D-LA, and pro-inflammation cytokines (IL-1β, TNF-α, and IL-6), while were negatively associated with weight gain, TP, ALB, tight junction proteins (ZO-1, occludin, and claudin-1), MUC2, SIgA, and IL-10. However, the *Lactobacillus* was positively associated with weight gain, TP, ALB, tight junction proteins (ZO-1, occludin, and claudin-1), MUC2, SIgA, and IL-10, but was significantly negatively correlated with fecal water content, LPS, IL-1β, and TNF-α. The relative abundance of *Muribaculum* was positively associated with occludin and claudin-1, while was significantly negatively correlated with D-LA, IL-1β, and TNF-α. The relative abundance of *Lachnospiraceae NK4A136 group* was negatively associated with ALB. The relative abundance of *Acinetobacter* was significantly negatively correlated with claudin-1. The relative abundance of *Lachnoclostridium* was positively correlated with IL-6, but was negatively correlated with SIgA (Figure 5).

## 4. Discussion

Antibiotic use over an extended period alters the balance of the intestinal microbiota, causes an imbalance in the gut microbiota, and can result in AAD (Overeem et al., 2008; Cui et al., 2020). Probiotics, on the other hand, regulate the gut microbiota, increase the integrity of the intestinal barrier, and enhance immunity (Wang X. et al., 2021). Therefore, finding complex probiotics that can better regulate gut microbiota is the first task of anti-AAD researchers.

Different types of probiotics exist, but the most common of them include Lactobacilli and Bifidobacteria, which are widely used in many functional foods and dietary supplements (Turroni et al., 2011; Xue et al., 2017). Studies have demonstrated that *Bifidobacterium lactis* XLTG11 can regulate the secretion of inflammatory cytokines and gut microbiota activities. Additionally, it produces substantial amounts of acetic, propionic, and butyric acid, which lower the intestinal pH, inhibit the proliferation of harmful bacteria, and strengthen gut protection, thereby improving AAD (Wang N. et al., 2021; Xu et al., 2022; Ma W. et al., 2023; Ma Y. et al., 2023). Additionally, *Lactobacillus casei* Zhang was established to regulate tight-junction proteins and inflammatory factor expression, alleviating gut microbiota disorders (Wang et al., 2019; Li X. et al., 2021). In addition, it also enhanced immunity by increasing the production of IgA and IFN-γ (Wang et al., 2013), and induced gut mucosal responses by promoting the production of SIgA (Ya et al., 2008). Studies have shown that *Lactobacillus plantarum* CCFM8661 can regulate the diversity and composition of the gut microbiota, alleviate the pathological damage to the colon and ileum, increase jejunum villus height-crypt depth ratio (Tian et al., 2012; Wang et al., 2019; Yu et al., 2021). *Lacticaseibacillus rhamnosus* Probio-M9 is a novel probiotic strain isolated from human breast milk of healthy women (Zhang et al., 2022). Oral Probio-M9 administration regulated the stability of the gut microbiota (Zheng et al., 2021). Probio-M9 has demonstrated excellent tolerance to gastrointestinal digestive fluids. Its survival rate after being subjected to artificial gastric fluid for 3 h is 83.72%, and after being subjected to artificial intestinal fluid for 11 h, it is 78.33%. Even when exposed to a pH of 2.5, Probio-M9 can pass through the stomach and reach the intestines to exert its beneficial effects (Liu et al., 2020). Furthermore, Probio-M9 can improve the intestinal environment by increasing the diversity of gut microbiota and regulating metabolic pathways (Xu et al., 2021). Furthermore, research has demonstrated that the symbiotic relationship between Lactobacillus and Bifidobacterium can synergistically enhance the production of metabolites, bolster their antibacterial capabilities, suppress the growth of pathogenic microorganisms, reinforce the integrity of the mucosal barrier, and promote the upregulation of immune responses (Chang et al., 2017). These studies showed that single species can regulate immunity and gut through different mechanisms, and we speculated that the combined use of multiple strains can enhance the immunomodulatory effects and protect the intestinal barrier function. In this experiment, *Bifidobacterium lactis* XLTG11, *Lactobacillus casei* Zhang, *Lactobacillus plantarum* CCFM8661, and *Lactobacillus rhamnosus* Probio-M9 were mixed at a ratio of 1:1:1:1 to prepare the complex probiotics utilized to study their alleviative effect on ampicillin-induced AAD.

Commonly used indicators to assess the severity of diarrhea include body weight gain, fecal water content, and fecal consistency (Hu et al., 2020; Ma Y. et al., 2023). *Bifidobacterium animalis* subsp. *lactis* XLTG11 not only significantly improved the diarrhea symptoms of AAD model mice, but also promoted the growth of mice (Ma Y. et al., 2023), which was consistent with our results. LPS and D-LA are important indicators to detect the degree of intestinal damage and permeability (Li et al., 2020; Ma Y. et al., 2023). Our results showed that the high-dose of the complex probiotics reduced the contents of LPS and D-LA to decrease intestinal permeability, which was similar to the results of Xu et al. (2022). TP can be divided into albumin and globulin. Serum protein can maintain the normal colloid osmotic pressure and PH of the blood, transport various metabolites, and regulate immune function. The results we obtained showed that the complex probiotics increased the levels of TP and ALB in the serum. These findings are similar to those of Jiang et al., who found that *Smilax glabra* extract elevated the levels of TP and ALB in antibiotic-associated diarrhea mice, and repaired the damage of the intestinal immune barrier (Jiang et al., 2022).

A primary function of the intestinal epithelium is to form a biological barrier that prevents the invasion of pathogenic antigens and maintains intestinal homeostasis (Feng et al., 2019; Jiang et al., 2021). Tight junctions are the main connection type between intestinal epithelial cells (Peterson and Artis, 2014). ZO-1, occludin, and claudin-1 are the major intestinal barrier proteins. MUC2 participates in the synthesis and secretion of goblet cells, which affect the permeability and integrity of the intestinal mucosa (Zhang et al., 2018; Zhuang et al., 2019). Lack of tight junction protein and MUC2 can induce intestinal epithelial dysfunction, increase intestinal barrier permeability, and increase the entry of harmful bacteria, which can lead to inflammation (Furuse et al., 1993; Van der Sluis et al., 2006; Al-Sadi et al., 2011; Pope et al., 2014; Kuo et al., 2021; Yao D. et al., 2021). Our results showed that the expression levels of ZO-1, claudin-1, occludin, and MUC2 in the colon of the MC group were decreased, indicating that ampicillin caused impairment of intestinal barrier function. However, the supplementation with complex probiotics improved intestinal epithelial permeability. In addition, studies have shown that *Lactobacillus plantarum* can increase the expression levels of ZO-1, occludin, and claudin-1, strengthen epithelial defense functions, and reduce intestinal mucosal permeability (Wang et al., 2018), *Bacteroides fragilis* can increase the expression levels of MUC2 and maintain the integrity of the intestinal barrier, alleviating symptoms of AAD in mice (Zhang et al., 2018), which are consistent with our results.

Antibiotic-associated diarrhea occurrence is accompanied by systemic inflammation (Ling et al., 2015). IL-6 can induce the differentiation of B cells. IL-6 promotes host defense through the stimulation of acute phase responses, immune reactions, and hematopoiesis (Popko et al., 2010; Tanaka et al., 2014). IL-1β is a major pro-inflammatory cytokine, it will aggravate the damage during chronic disease and acute tissue damage (Lopez-Castejon and Brough, 2011). TNF-α is an inflammatory cytokine, which can cause inflammation or cell apoptosis. TNF-α induces the release of a large number of cytokines, including IL-6, IL-8, and IL-1β by stimulating macrophages (Idriss and Naismith, 2000; Ma et al., 2016). IL-10 is a cytokine with a significant anti-inflammatory effect. It inhibits the release of pro-inflammatory factors, and plays an essential role in maintaining gastrointestinal homeostasis (Iyer and Cheng, 2012; Li et al., 2014). The results of this experiment showed that complex probiotics significantly reduced the levels of IL-6, IL-1β, and TNF-α while increasing the level of IL-10 in the colon. Therefore, complex probiotics have a good anti-inflammatory effect in AAD. Studies have shown that echinacin attenuated LPS-induced secretion and mRNA expression of TNF-α and IL-6, enhanced the secretion and mRNA expression of IL-10, and weakened the inflammatory response, which is consistent with our previous results (Li et al., 2018). SIgA is the main component of the mucosal defense system of the body and plays a decisive role in the first line of defense against diseases (Kumar et al., 2020; Chen S. et al., 2022). Our present results showed that complex probiotics increased the SIgA level in the colon tissue, which was similar to the results of Peng C. et al. (2022).

In this study, we further analyzed the diversity and richness of the intestinal microorganisms at the phylum and genus levels. At the phylum level, *Bacteroidetes*, *Proteobacteria*, and *Firmicutes* were the dominant phyla (Shin et al., 2015; Guan et al., 2018). *Bacteroides* and *Firmicutes* were also established to be abundant in the intestinal tract of healthy people (Schultz et al., 2017). An increase in the relative abundance of *Proteobacteria* led to a decrease in short-chain fatty acid (SCFA) production (Meng et al., 2020), which indicates a risk of infection and metabolic disorders (Shao et al., 2020). Our results showed that ampicillin administration transformed the composition of the gut microbiota in the examined groups, leading to *Proteobacteria* overgrowth and restricted growth of *Bacteroidetes.* Notably, the complex probiotic treatment reversed these changes, which was similar to the results obtained by Wang N. et al. (2021). At the genus level, *Bacteroides* is a Gram-negative, obligate anaerobic bacteria that controls lymphocyte and cytokine expression and suppresses inflammation (Tan et al., 2019). *Muribaculateae* can reduce cholesterol content and prevent excessive cholesterol levels by regulating intestinal microbiota and its metabolites (Li et al., 2022). *Klebsiella* is the pathogen of antibiotic-associated hemorrhagic colitis (Högenauer et al., 2006), it was positively correlated with inflammatory factors such as TNF-α and IL-6 (Liang et al., 2022). Lactic acid and other metabolites produced by the *Lactobacillus* living in the intestine can kill pathogenic microorganisms. In addition, such microbial metabolites also regulate gut microbiota diversity, enhance immunity, improve intestinal function, and maintain normal intestinal homeostasis (Zafar and Saier, 2020). The results of this experiment showed that the treatment with complex probiotics regulated the abundance of *Bacteroides*, *Muribaculateae*, *Klebsiella*, *Lactobacillus*, and *Parabolides*, which was similar to earlier results (Xu et al., 2022). All of the aforementioned experimental findings imply that complex probiotics may exert therapeutic effects in patients with diarrhea and gut microbiota imbalance caused by antibiotics.

This study used mice as the animal model for diarrhea because mice share a high genetic similarity with humans; mice have pure strains, which can reduce interference from different strains; mice are small and easy to breed indoors on a large scale. However, there are significant differences between mice and humans in anatomy and physiology. Moreover, the mice model cannot reflect how human lifestyle, diet, age, and other factors affect the gut microbiota and immune system. These limitations may reduce the generalizability and validity of the experimental results, making it hard to apply the research on complex probiotics for treating AAD to clinical practice. Therefore, future research should consider using animal models closer to humans or testing the findings of this study in clinical trials.

## 5. Conclusion

In summary, complex probiotics composed of *Bifidobacterium lactis* XLTG11, *Lactobacillus casei* Zhang, *Lactobacillus plantarum* CCFM8661, and *Lactobacillus rhamnosus* Probio-M9 could alleviate ampicillin-induced AAD in mice including protecting intestinal barrier function, regulating the secretion of proinflammatory factors, the gut microbiota diversity and composition. Our research will provide further evidence and theoretical support for the treatment of antibiotic-associated diarrhea with complex probiotics. In the future, it will be feasible to study the differential therapeutic efficacy among various combinations of microbial strains and explore which strains or combinations are more effective in treating AAD, thereby providing more robust evidence for their clinical application.

## Data availability statement

The datasets presented in this study can be found in online repositories. The names of the repository/repositories and accession number(s) can be found in the article/Supplementary material.

## Ethics statement

The animal study was reviewed and approved by all animal procedures were carried out by Heilongjiang University of Traditional Chinese Medicine’s Regulations on the Administration of Laboratory Animals, and the University’s Animal Ethics Committee approved all experiments (Ethic approval code: 2021121201).

## Author contributions

WL and SZ participated in the analysis of experimental data, analyzed and discussed the results, drew graphs, and wrote the paper. YW, HB, and SY conducted experiments, analyzed and discussed the results, and helped to revise the manuscript. LH and WM were mainly responsible for conceiving and designing the study, directing and supervising the experiment, and helping to revise the manuscript. All authors contributed to the article and approved the submitted version.

## Funding

This work was funded by Natural Science Foundation of Heilongjiang Province of China (LH2019H106), Heilongjiang Province Touyan Team.

## Conflict of interest

The authors declare that the research was conducted in the absence of any commercial or financial relationships that could be construed as a potential conflict of interest.

## Publisher’s note

All claims expressed in this article are solely those of the authors and do not necessarily represent those of their affiliated organizations, or those of the publisher, the editors and the reviewers. Any product that may be evaluated in this article, or claim that may be made by its manufacturer, is not guaranteed or endorsed by the publisher.
