# Supplementary figures and images for: Complex probiotics alleviate ampicillin-induced antibiotic-associated diarrhea in mice
Source: Front Microbiol. 2023 Apr 14;14:1156058. doi: 10.3389/fmicb.2023.1156058 (PMC10145528; doi:10.3389/fmicb.2023.1156058)

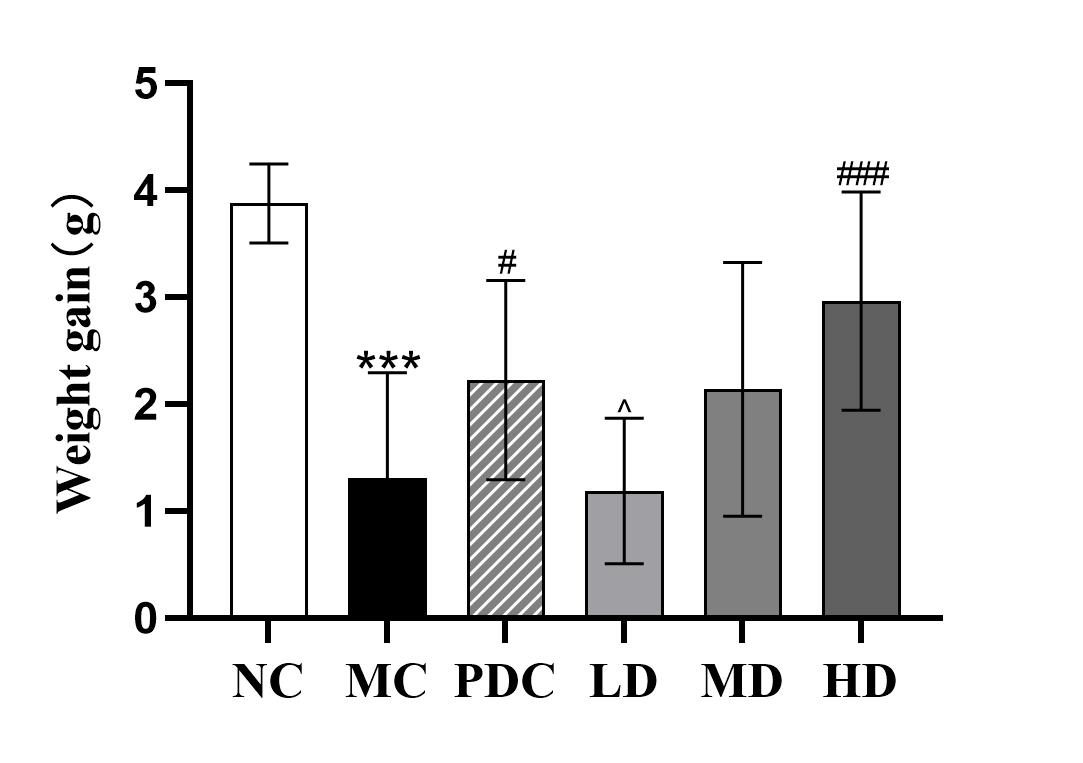

Supplement: Supplementary file 1 [file Data_Sheet_1.zip › Data Sheet 1/Figure 1A.jpg]

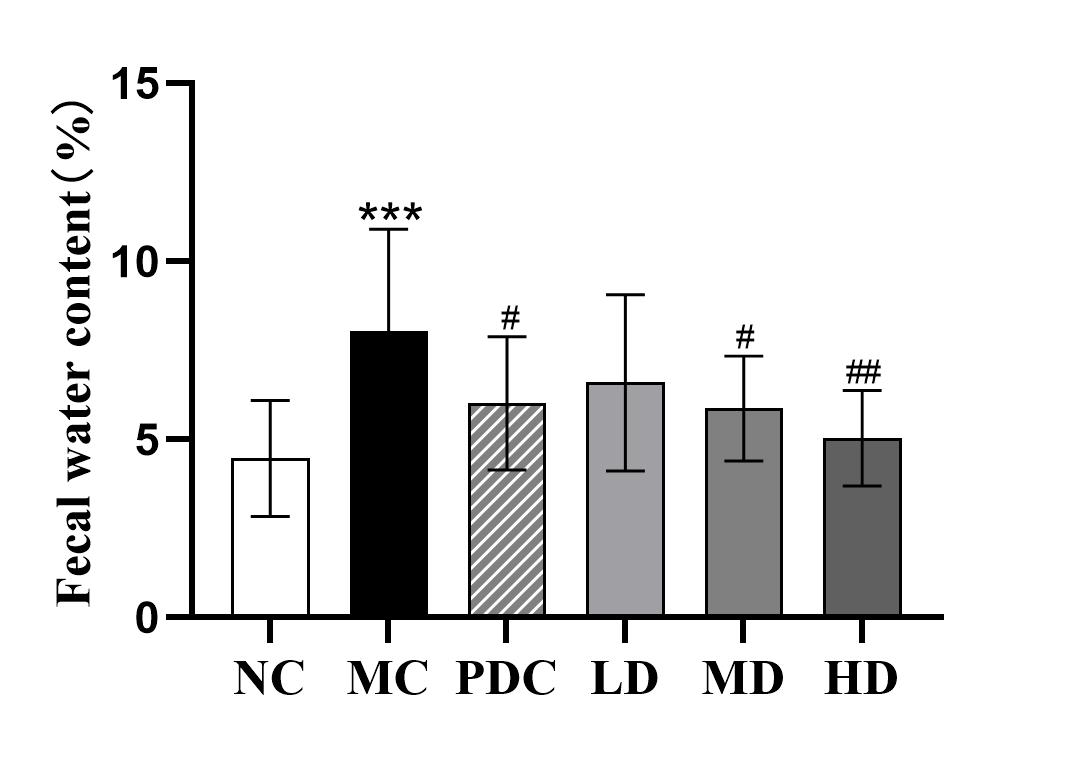

Supplement: Supplementary file 1 [file Data_Sheet_1.zip › Data Sheet 1/Figure 1B.jpg]

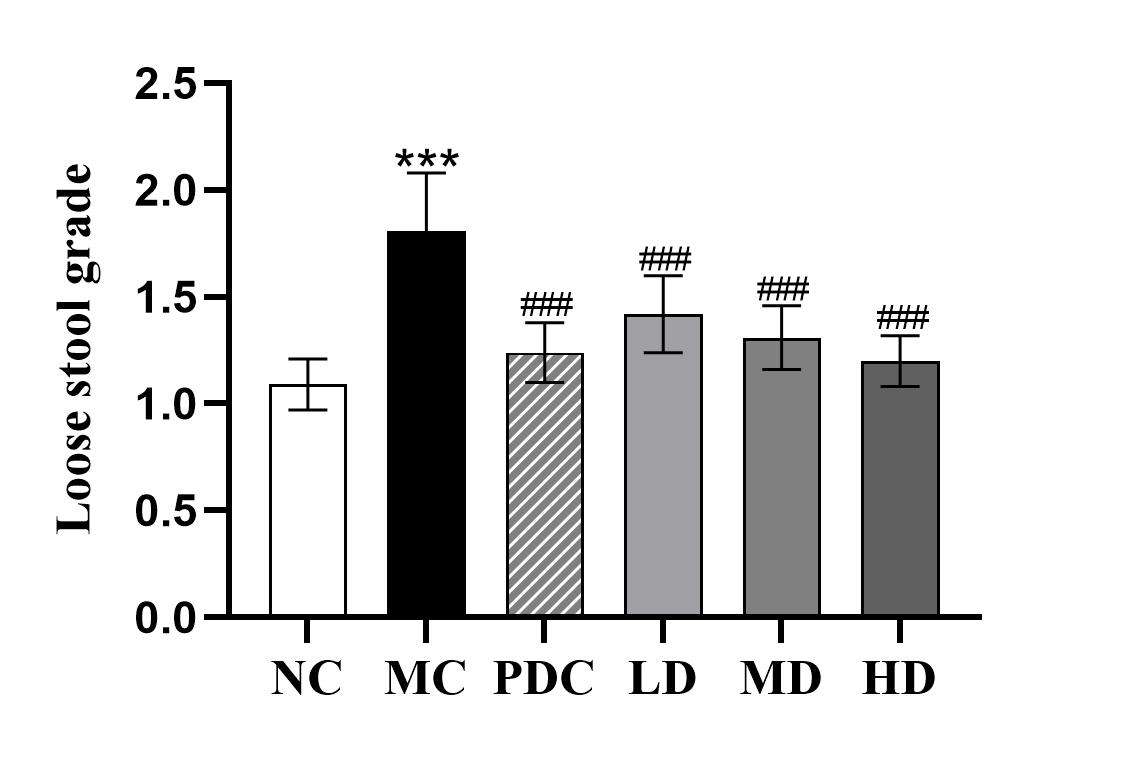

Supplement: Supplementary file 1 [file Data_Sheet_1.zip › Data Sheet 1/Figure 1C.jpg]

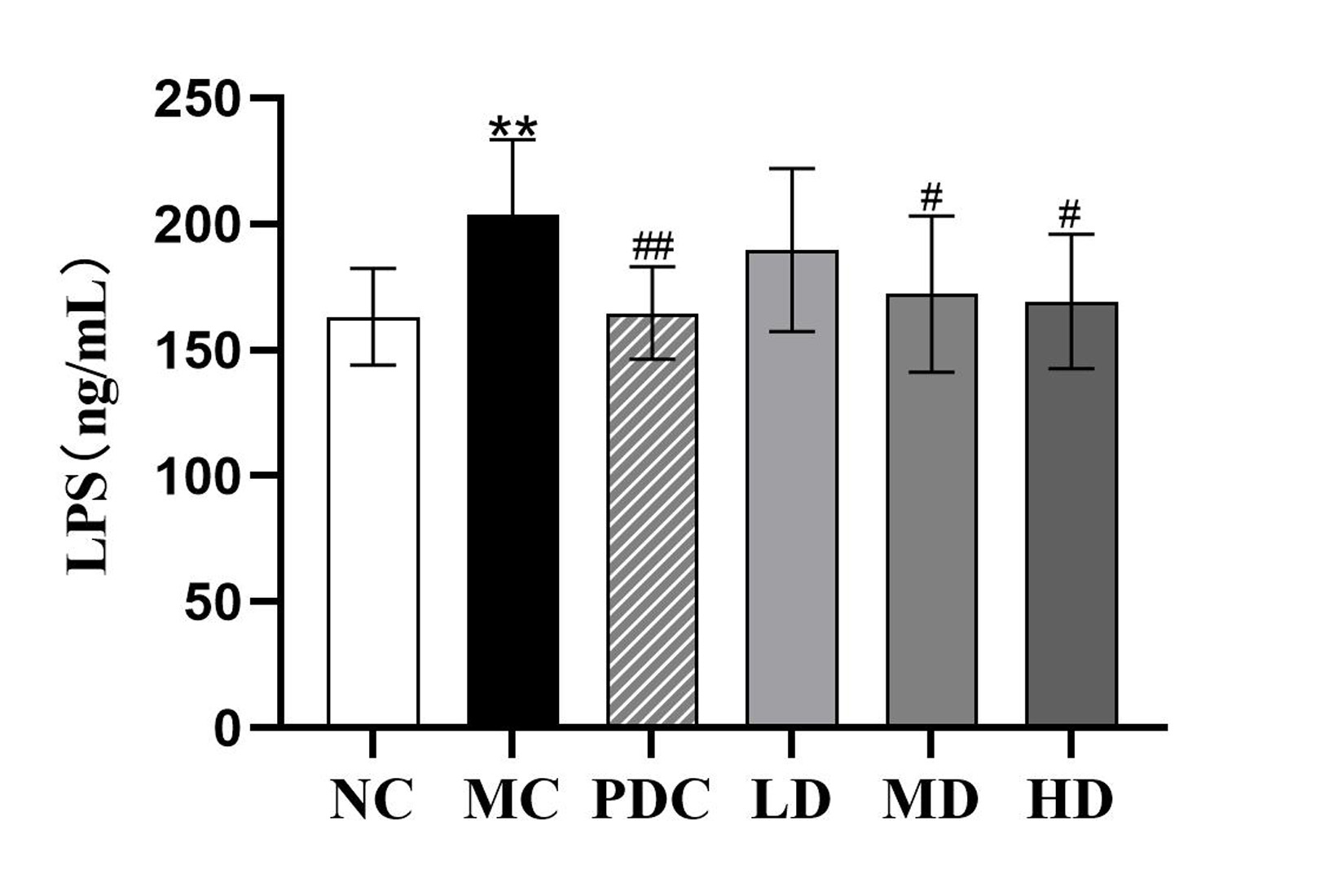

Supplement: Supplementary file 1 [file Data_Sheet_1.zip › Data Sheet 1/Figure 1D.jpg]

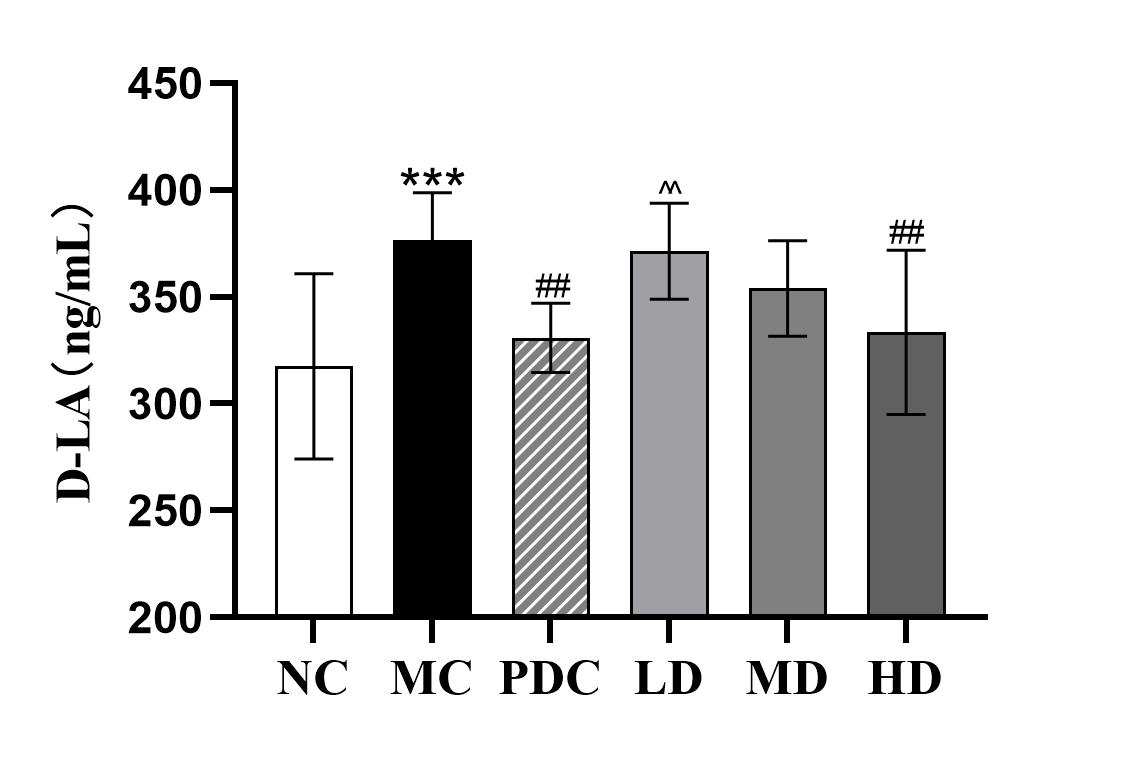

Supplement: Supplementary file 1 [file Data_Sheet_1.zip › Data Sheet 1/Figure 1E.jpg]

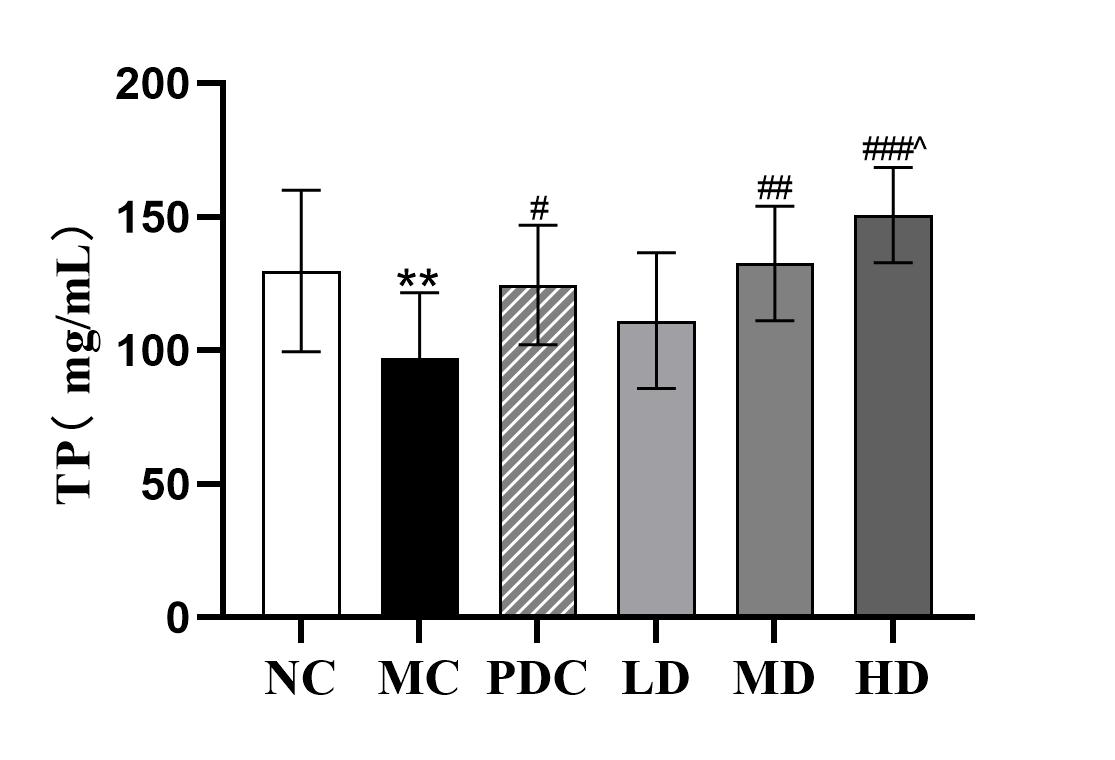

Supplement: Supplementary file 1 [file Data_Sheet_1.zip › Data Sheet 1/Figure 2A.jpg]

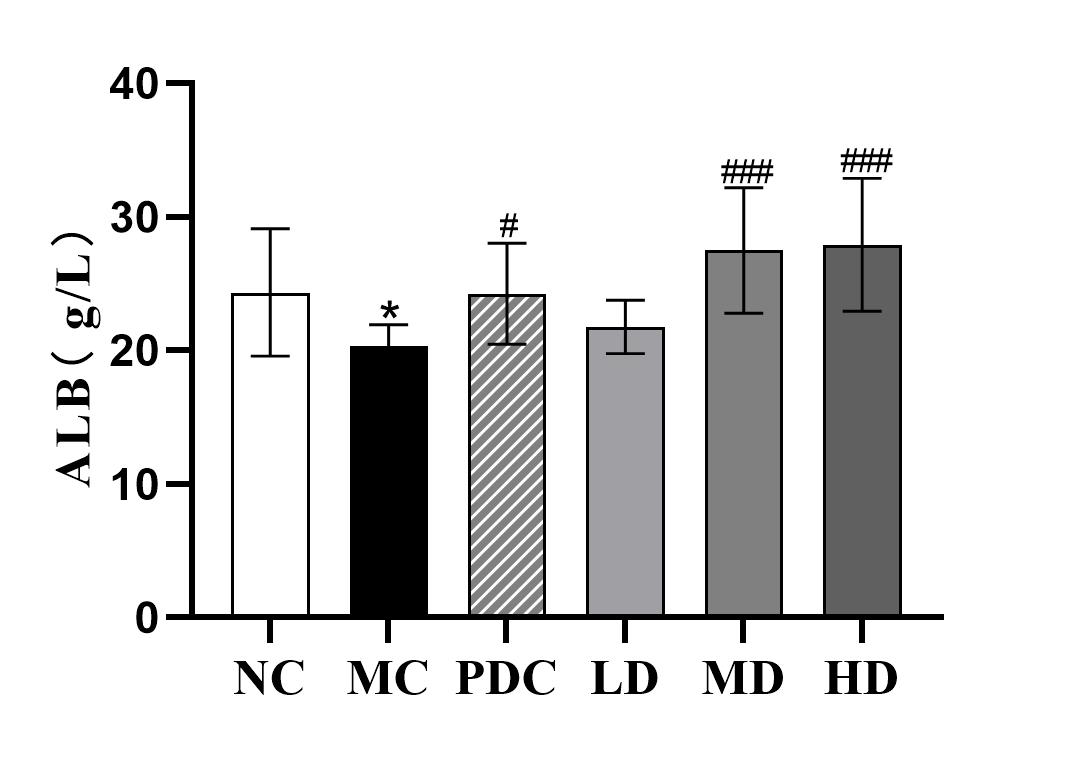

Supplement: Supplementary file 1 [file Data_Sheet_1.zip › Data Sheet 1/Figure 2B.jpg]

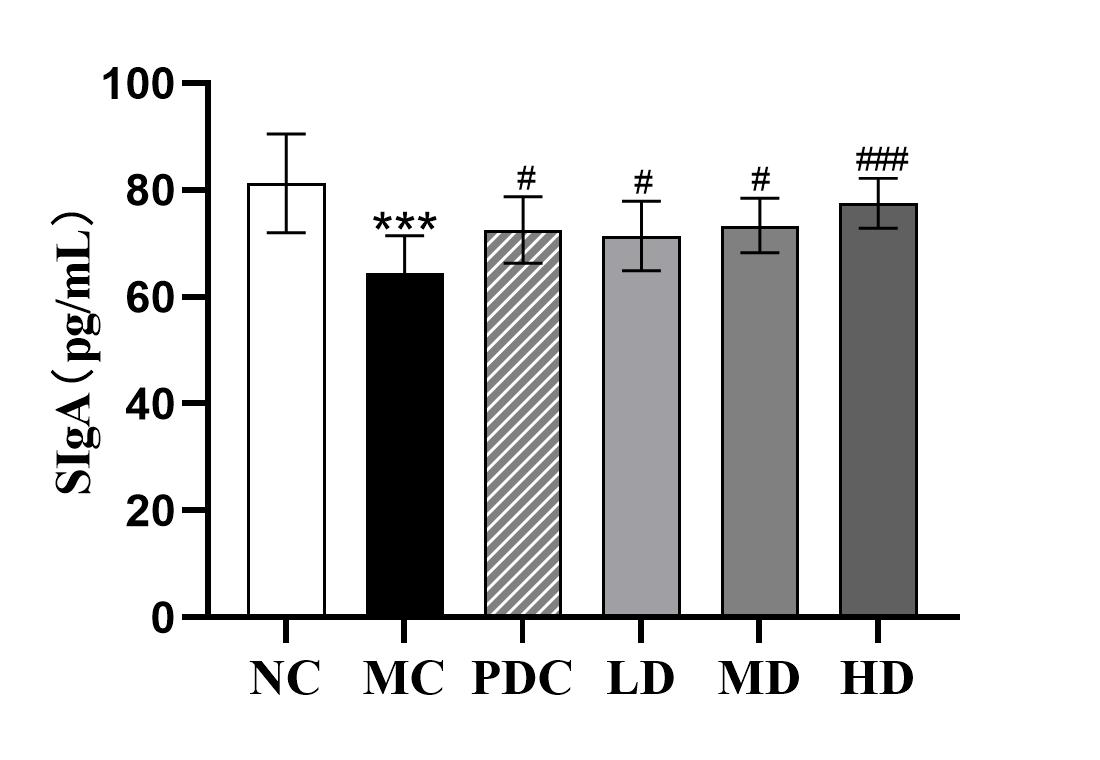

Supplement: Supplementary file 1 [file Data_Sheet_1.zip › Data Sheet 1/Figure 2C.jpg]

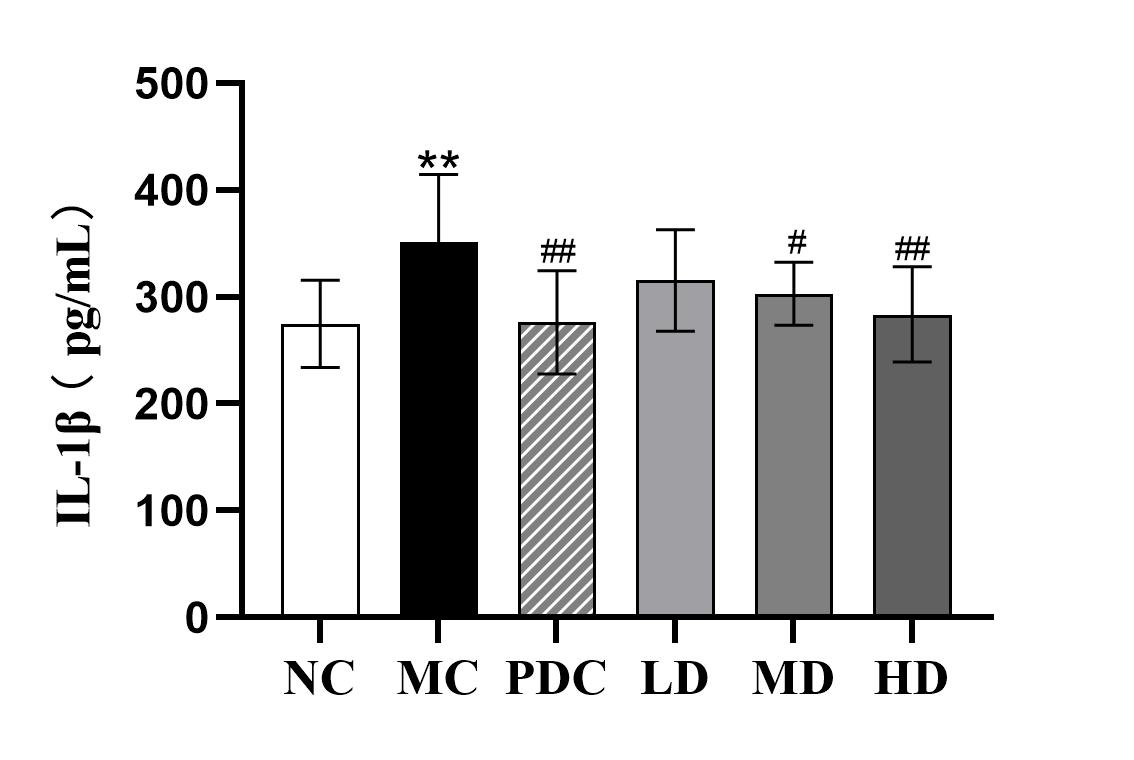

Supplement: Supplementary file 1 [file Data_Sheet_1.zip › Data Sheet 1/Figure 2D.jpg]

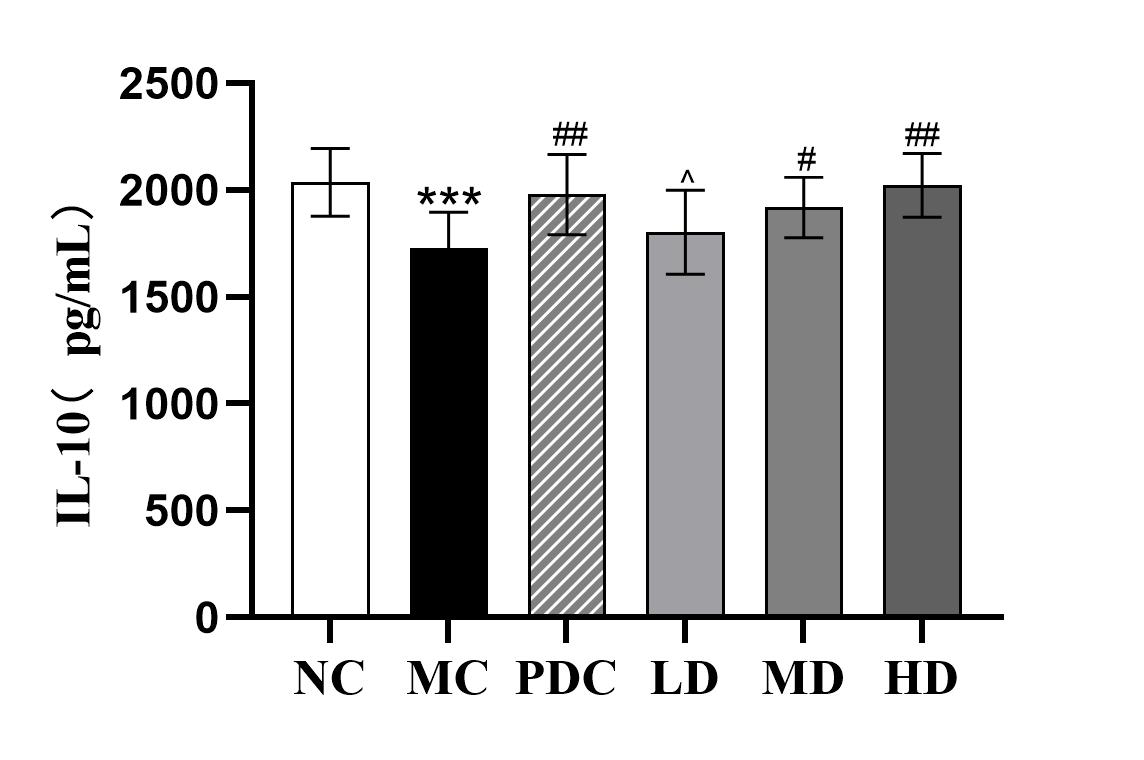

Supplement: Supplementary file 1 [file Data_Sheet_1.zip › Data Sheet 1/Figure 2E.jpg]

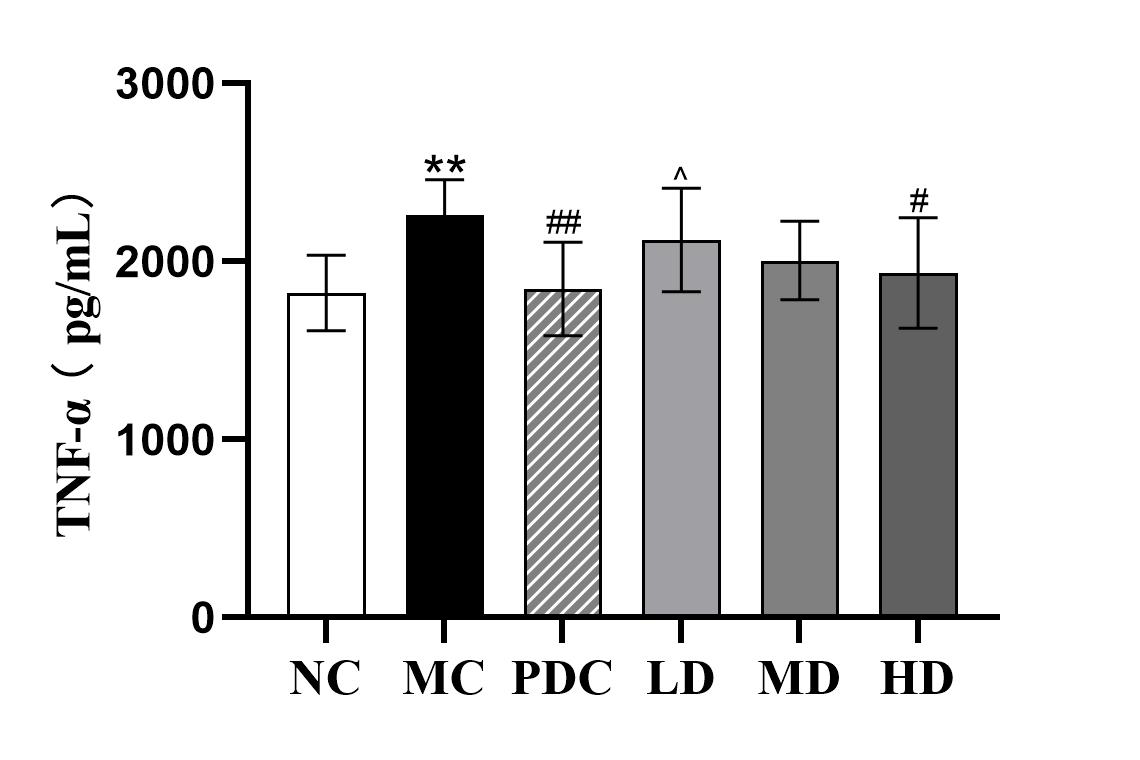

Supplement: Supplementary file 1 [file Data_Sheet_1.zip › Data Sheet 1/Figure 2F.jpg]

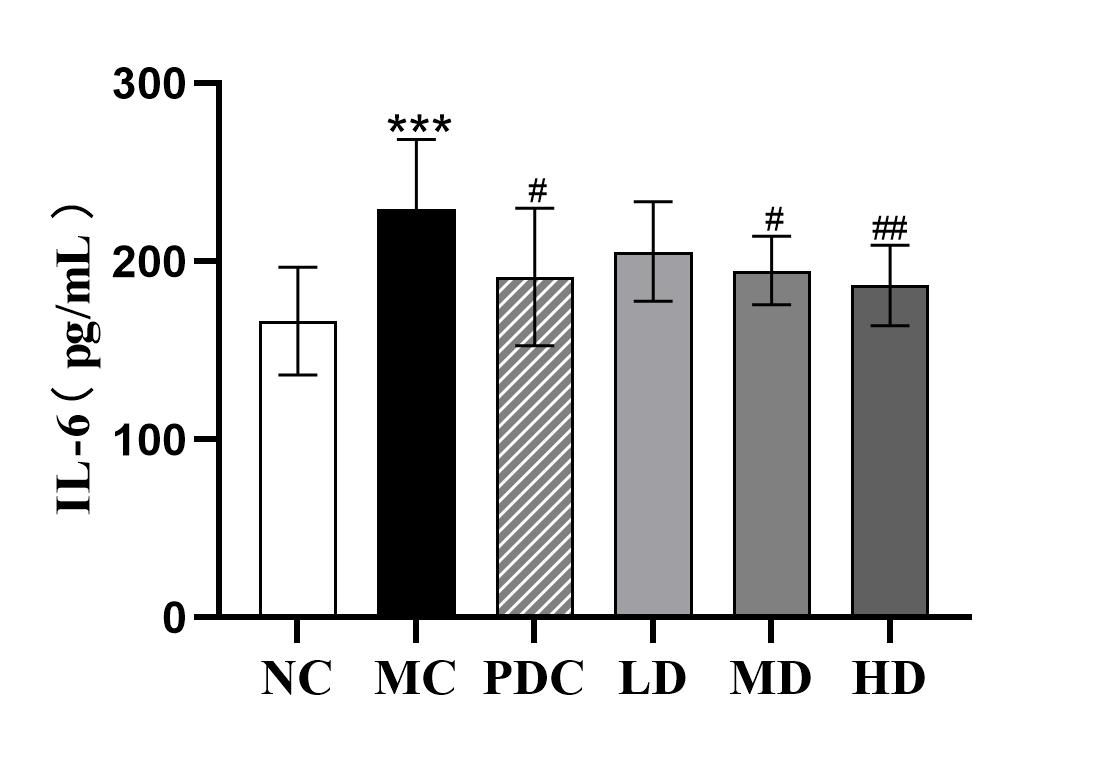

Supplement: Supplementary file 1 [file Data_Sheet_1.zip › Data Sheet 1/Figure 2G.jpg]

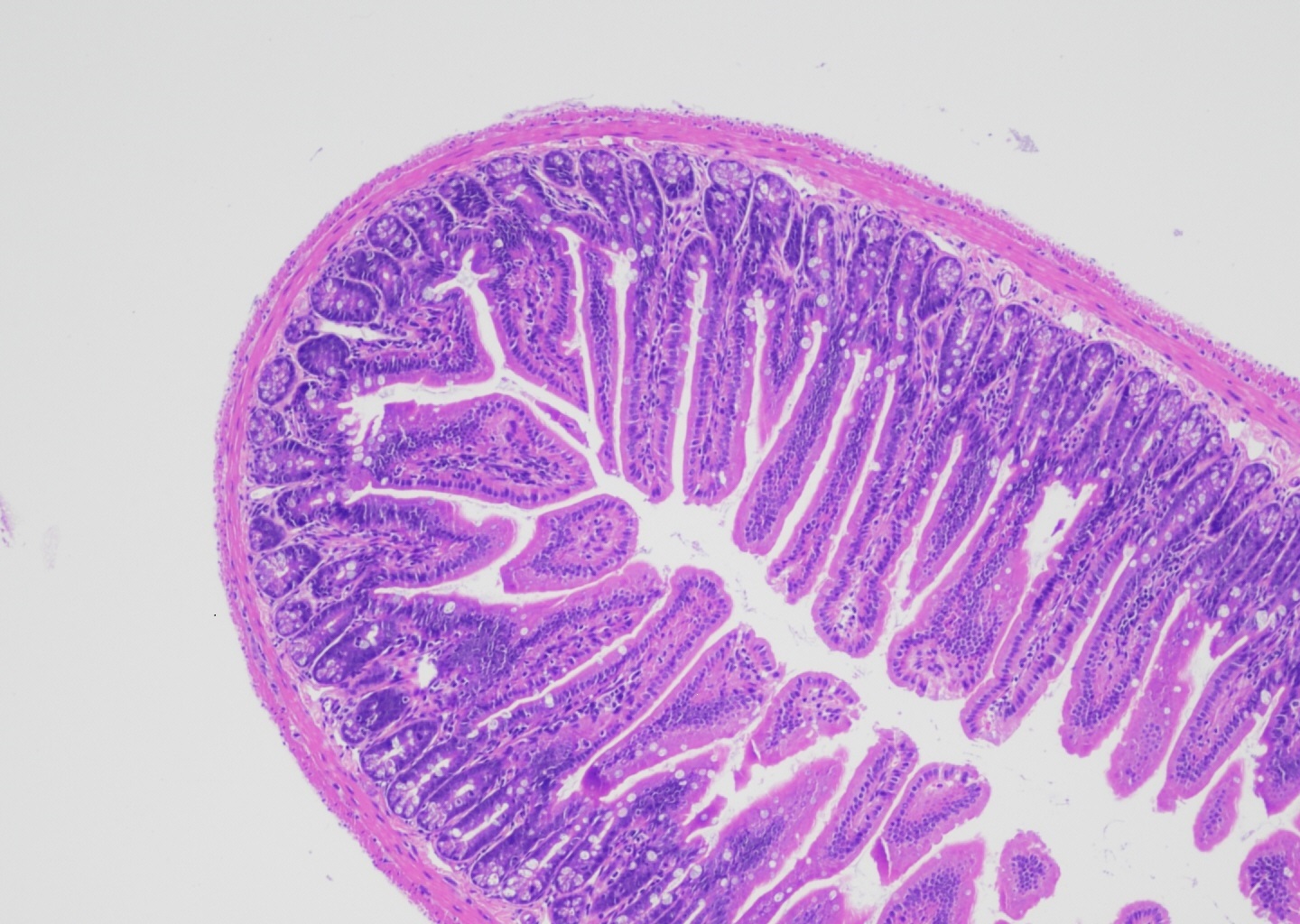

Supplement: Supplementary file 1 [file Data_Sheet_1.zip › Data Sheet 1/Figure 3A(HD).jpg]

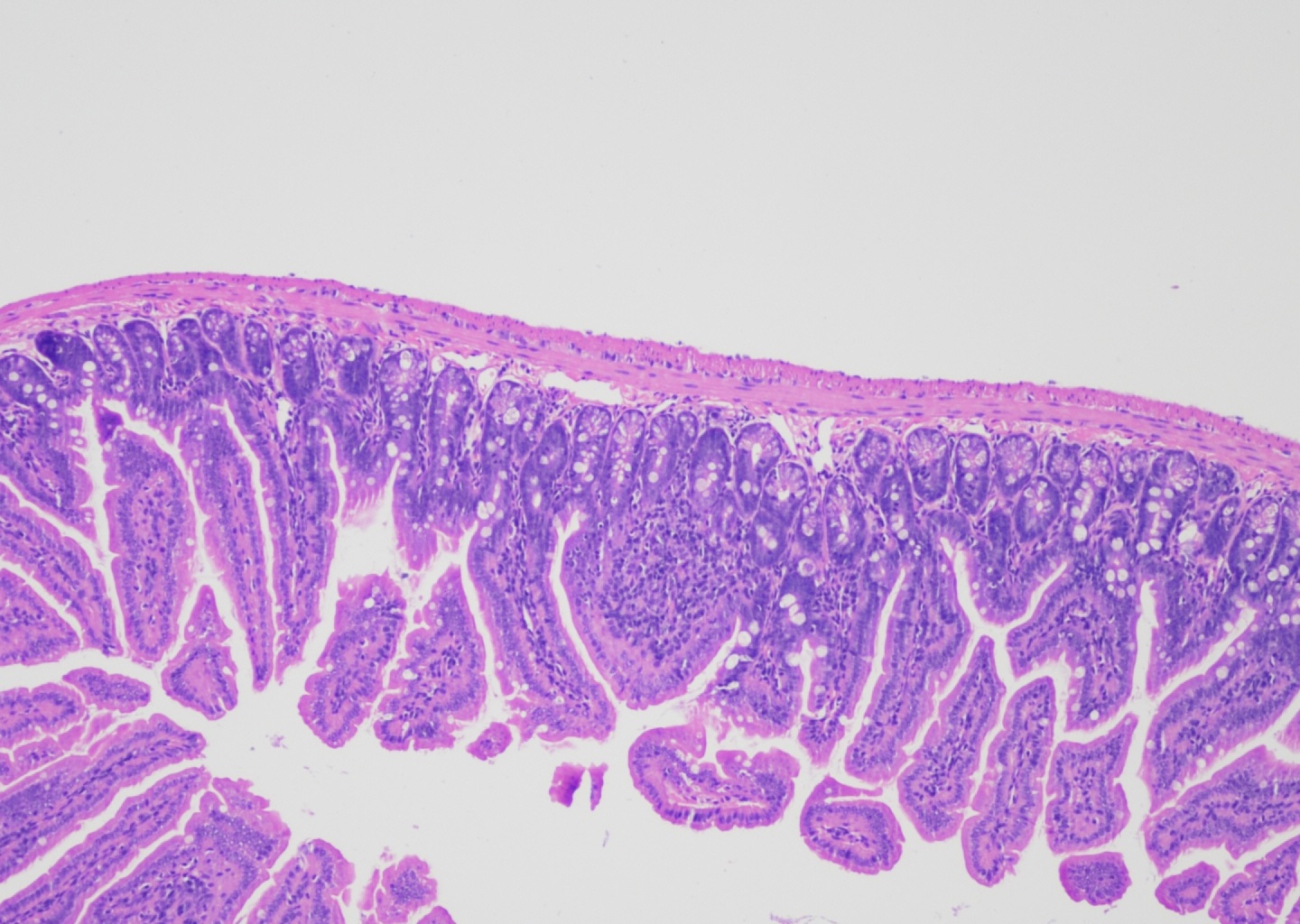

Supplement: Supplementary file 1 [file Data_Sheet_1.zip › Data Sheet 1/Figure 3A(LD).jpg]

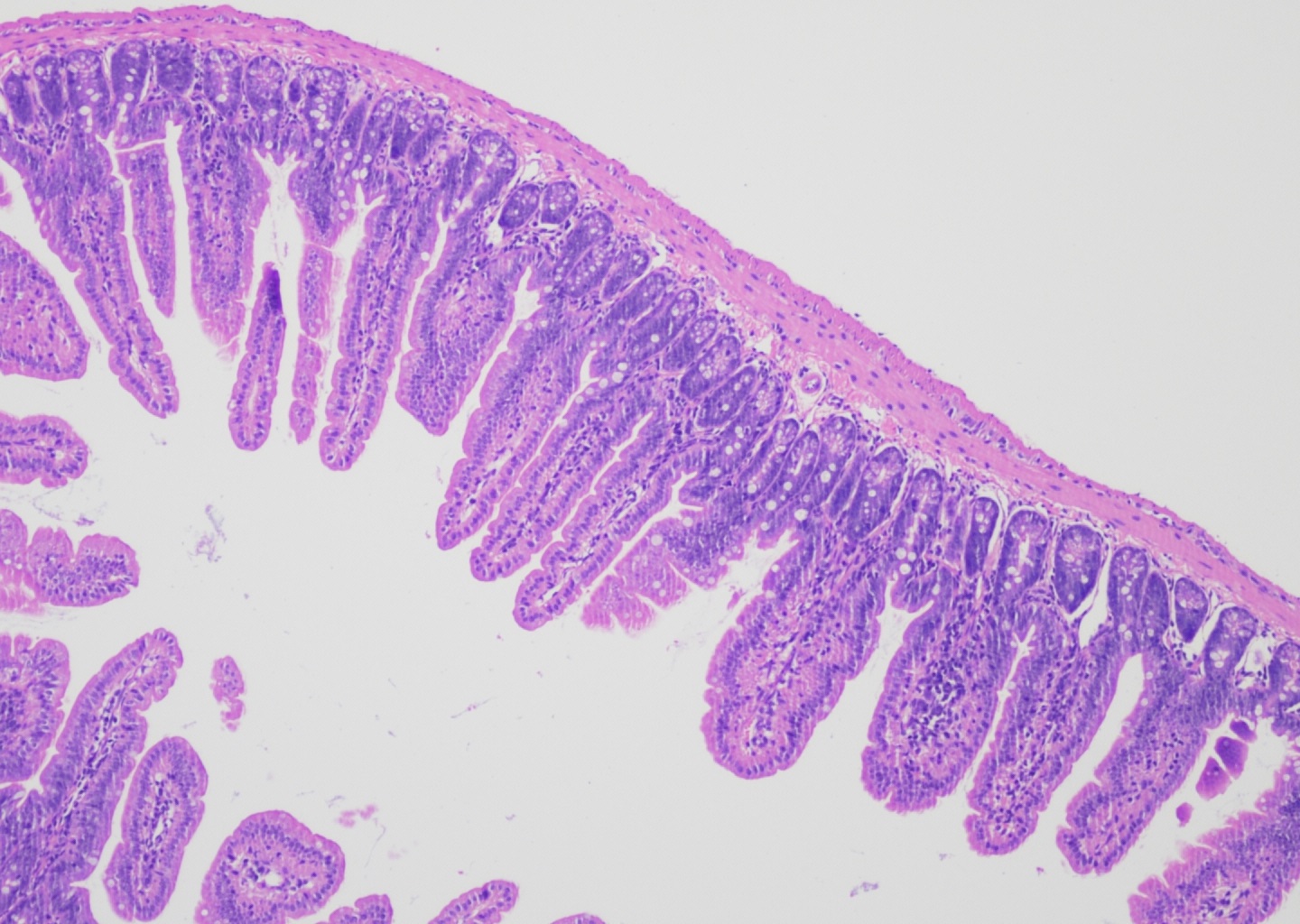

Supplement: Supplementary file 1 [file Data_Sheet_1.zip › Data Sheet 1/Figure 3A(MC).jpg]

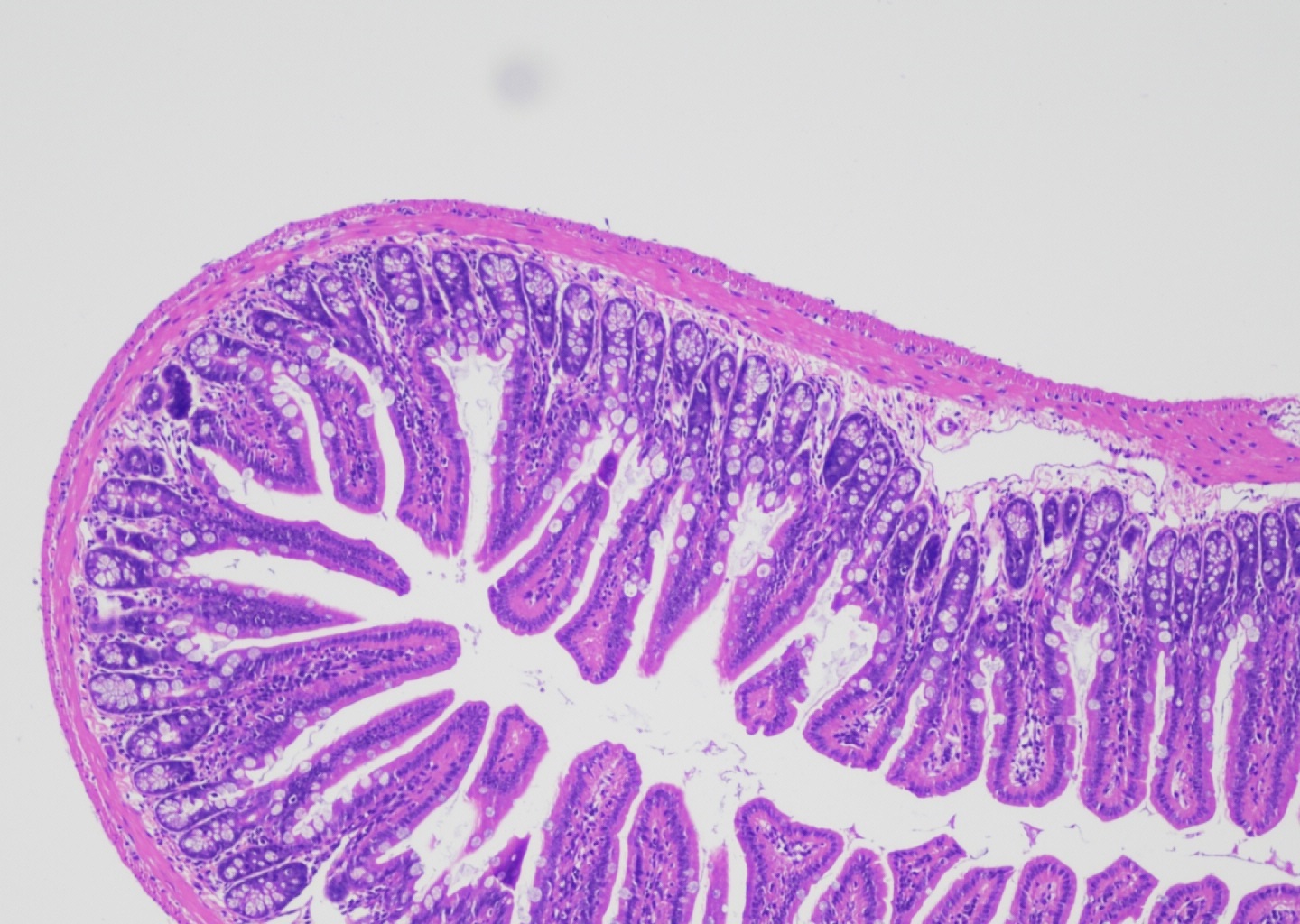

Supplement: Supplementary file 1 [file Data_Sheet_1.zip › Data Sheet 1/Figure 3A(MD).jpg]

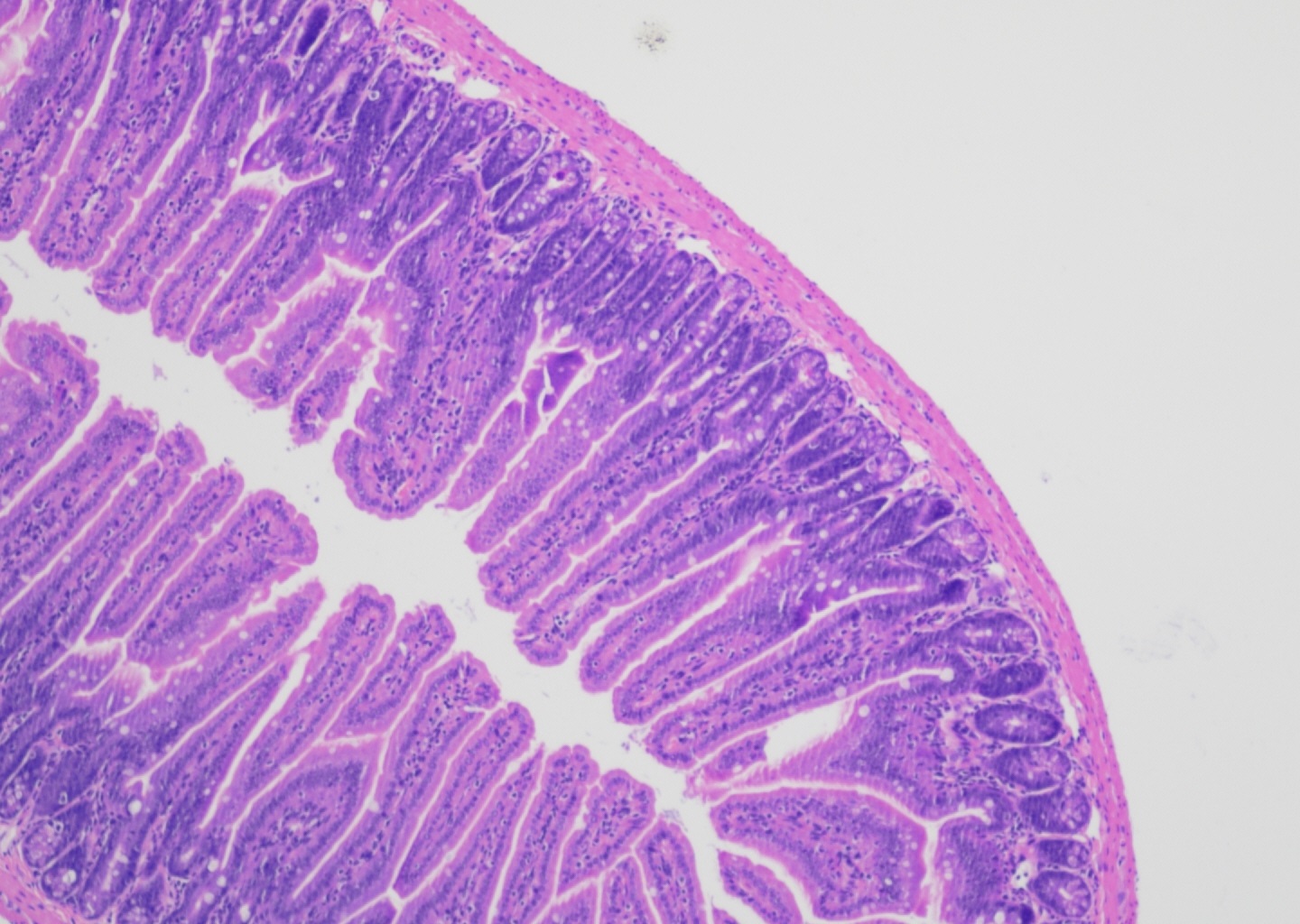

Supplement: Supplementary file 1 [file Data_Sheet_1.zip › Data Sheet 1/Figure 3A(NC).jpg]

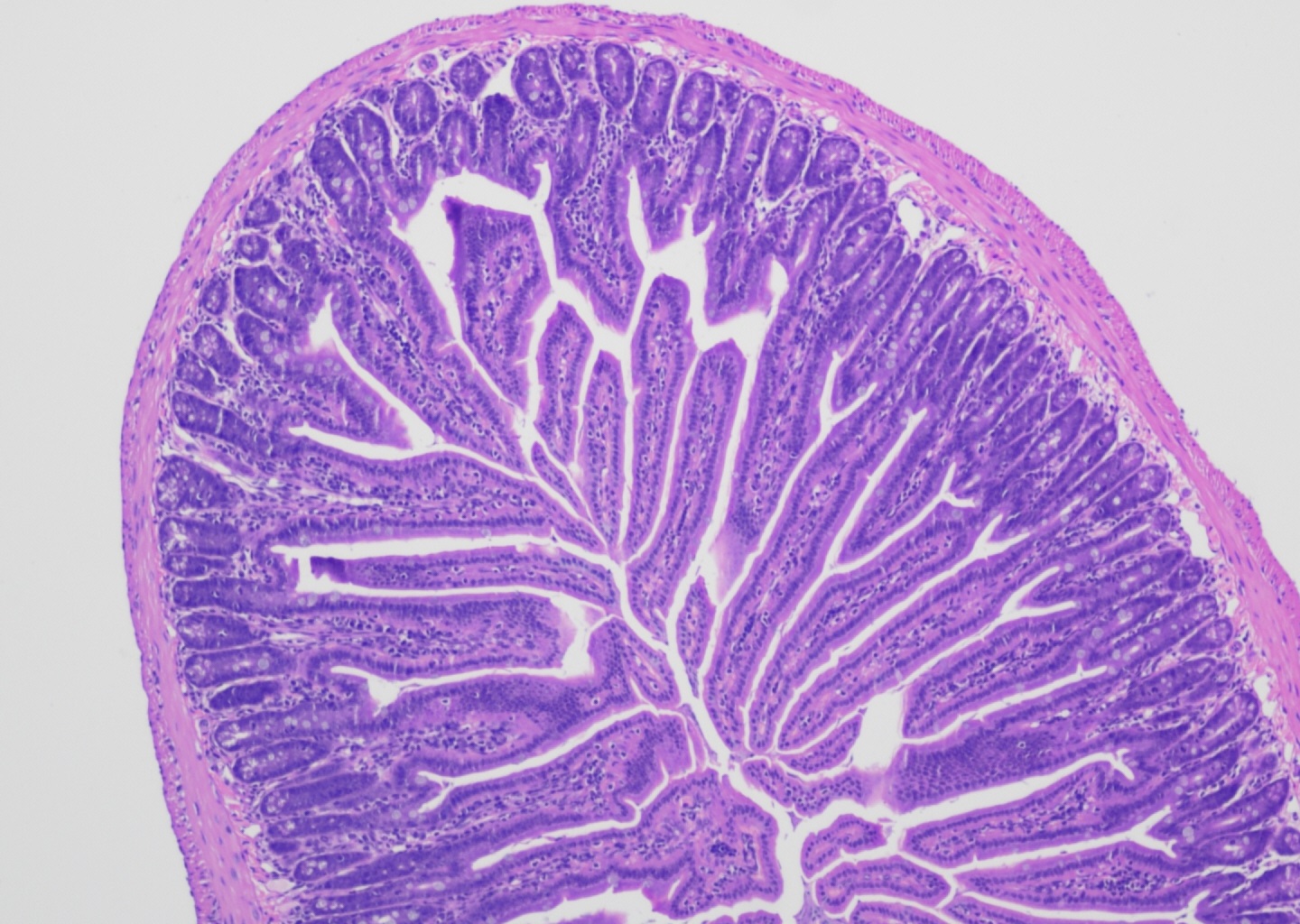

Supplement: Supplementary file 1 [file Data_Sheet_1.zip › Data Sheet 1/Figure 3A(PDC).jpg]

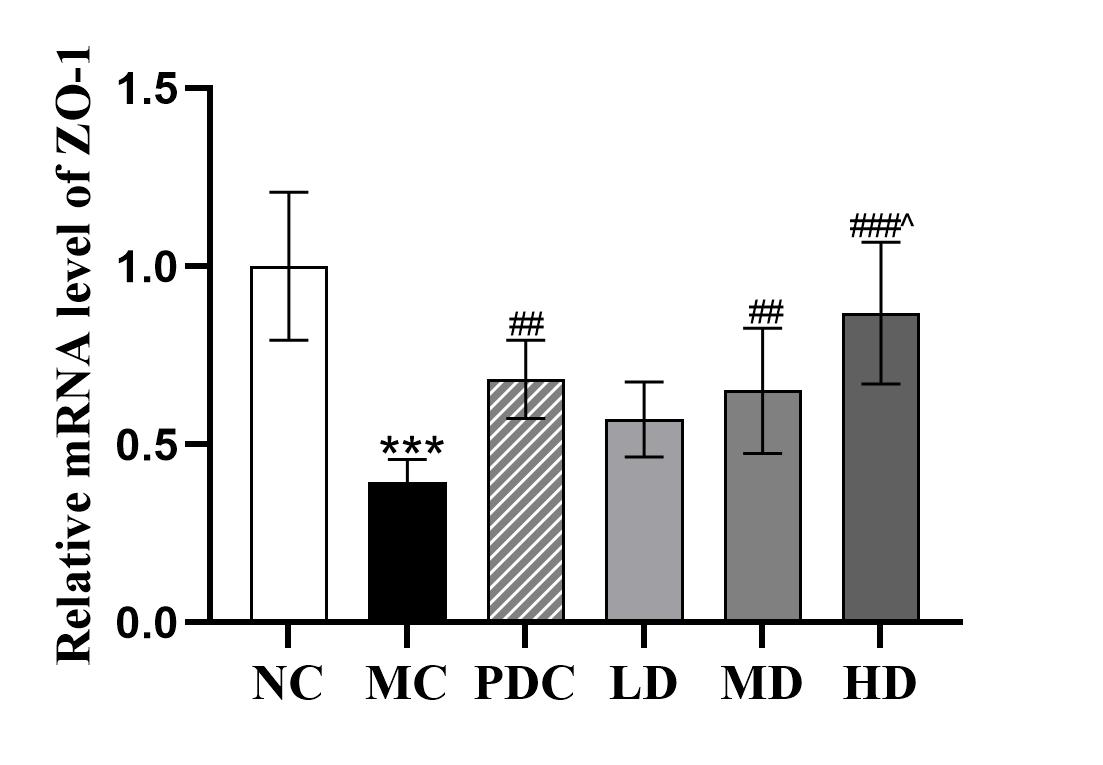

Supplement: Supplementary file 1 [file Data_Sheet_1.zip › Data Sheet 1/Figure 3B.jpg]

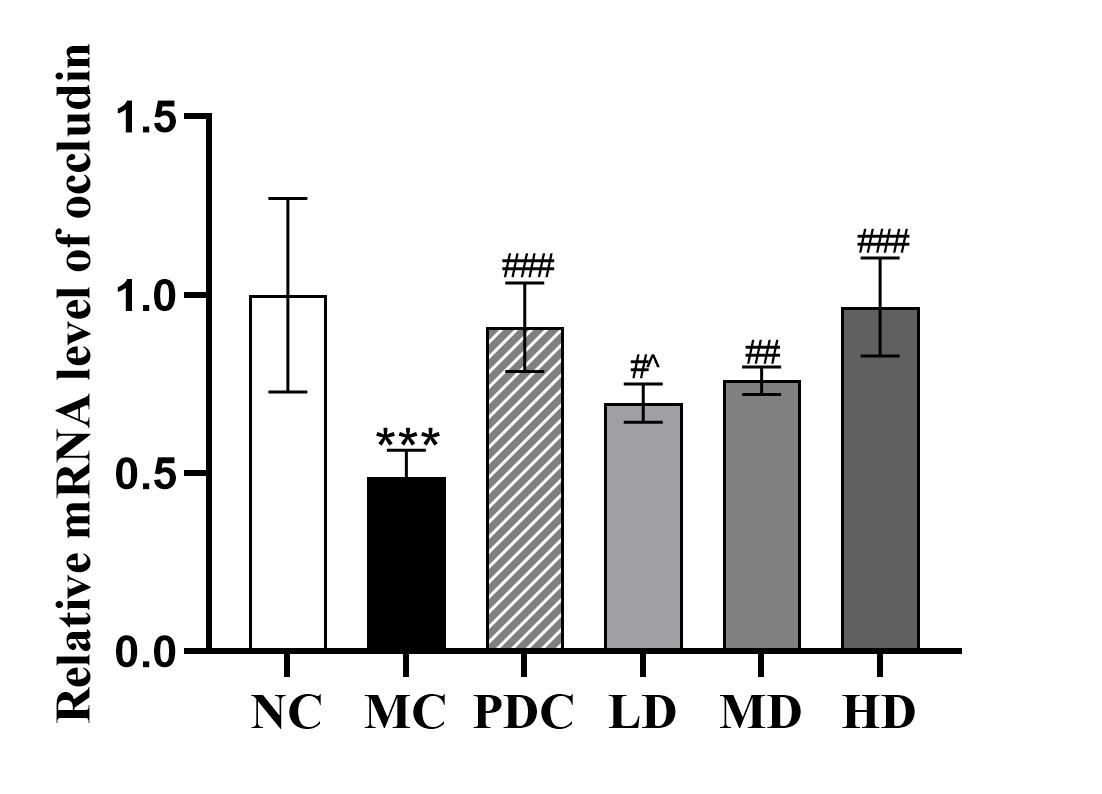

Supplement: Supplementary file 1 [file Data_Sheet_1.zip › Data Sheet 1/Figure 3C.jpg]

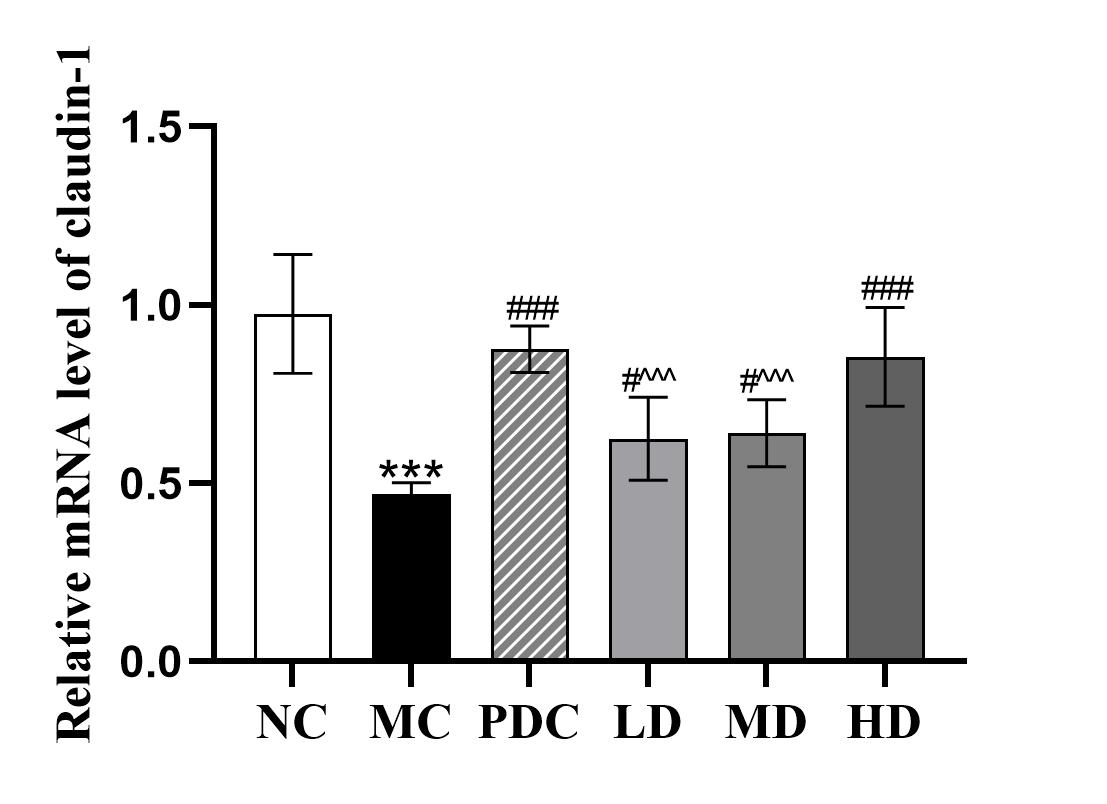

Supplement: Supplementary file 1 [file Data_Sheet_1.zip › Data Sheet 1/Figure 3D.jpg]

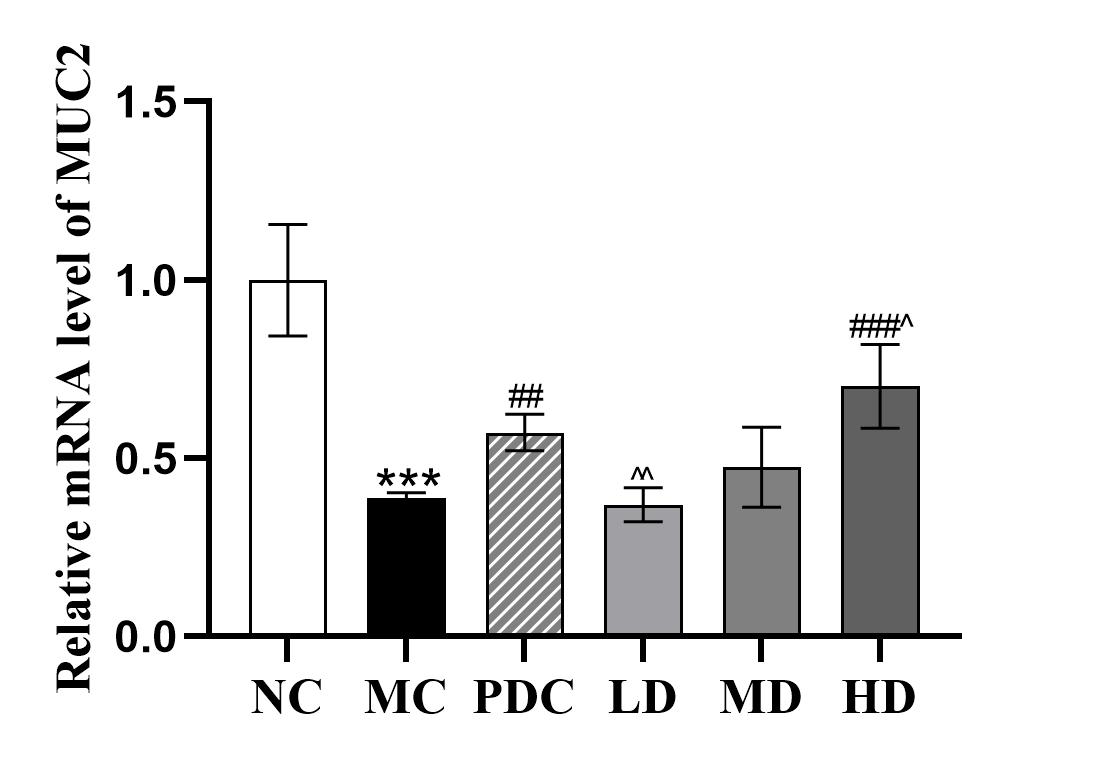

Supplement: Supplementary file 1 [file Data_Sheet_1.zip › Data Sheet 1/Figure 3E.jpg]

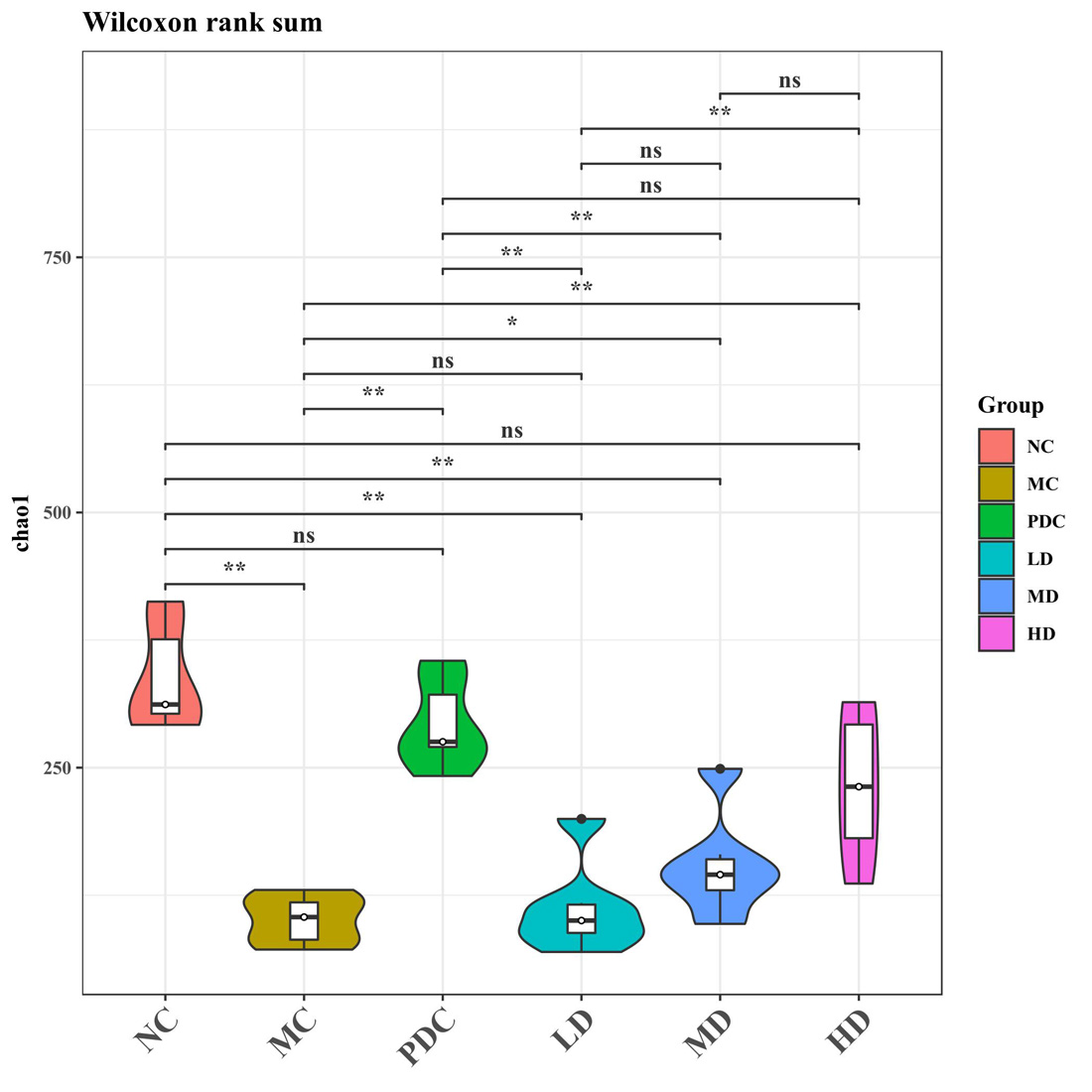

Supplement: Supplementary file 1 [file Data_Sheet_1.zip › Data Sheet 1/Figure 4A.jpg]

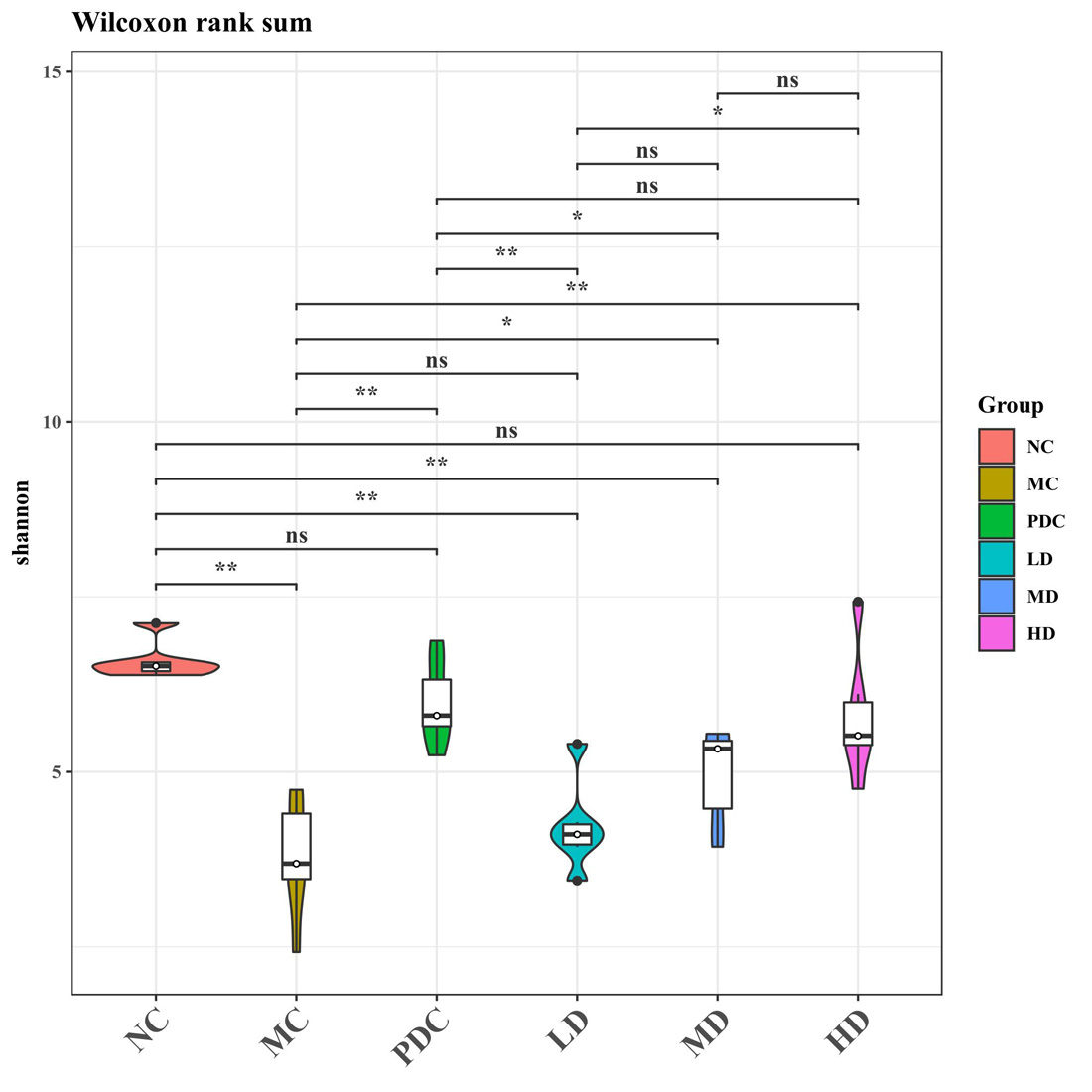

Supplement: Supplementary file 1 [file Data_Sheet_1.zip › Data Sheet 1/Figure 4B.jpg]

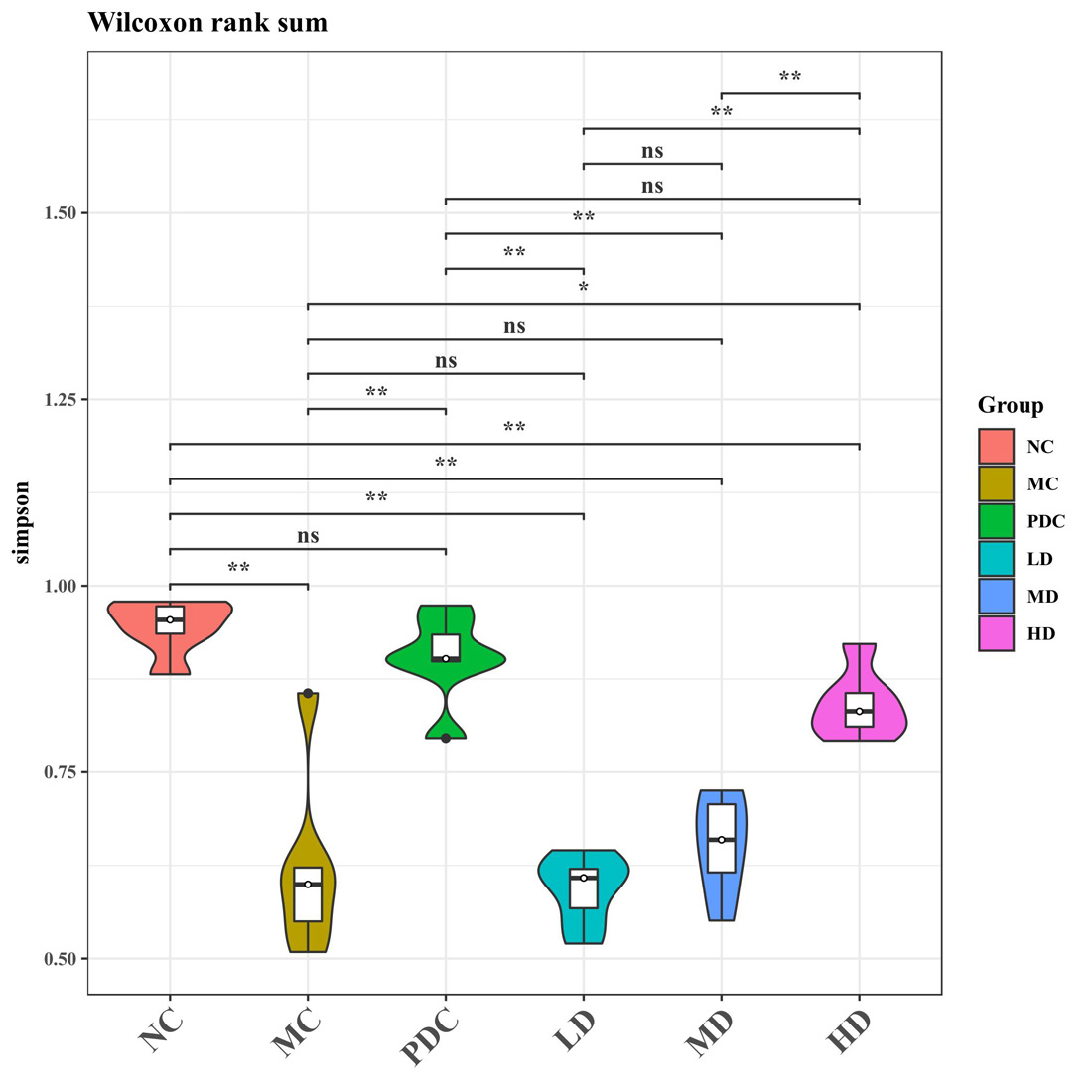

Supplement: Supplementary file 1 [file Data_Sheet_1.zip › Data Sheet 1/Figure 4C.jpg]

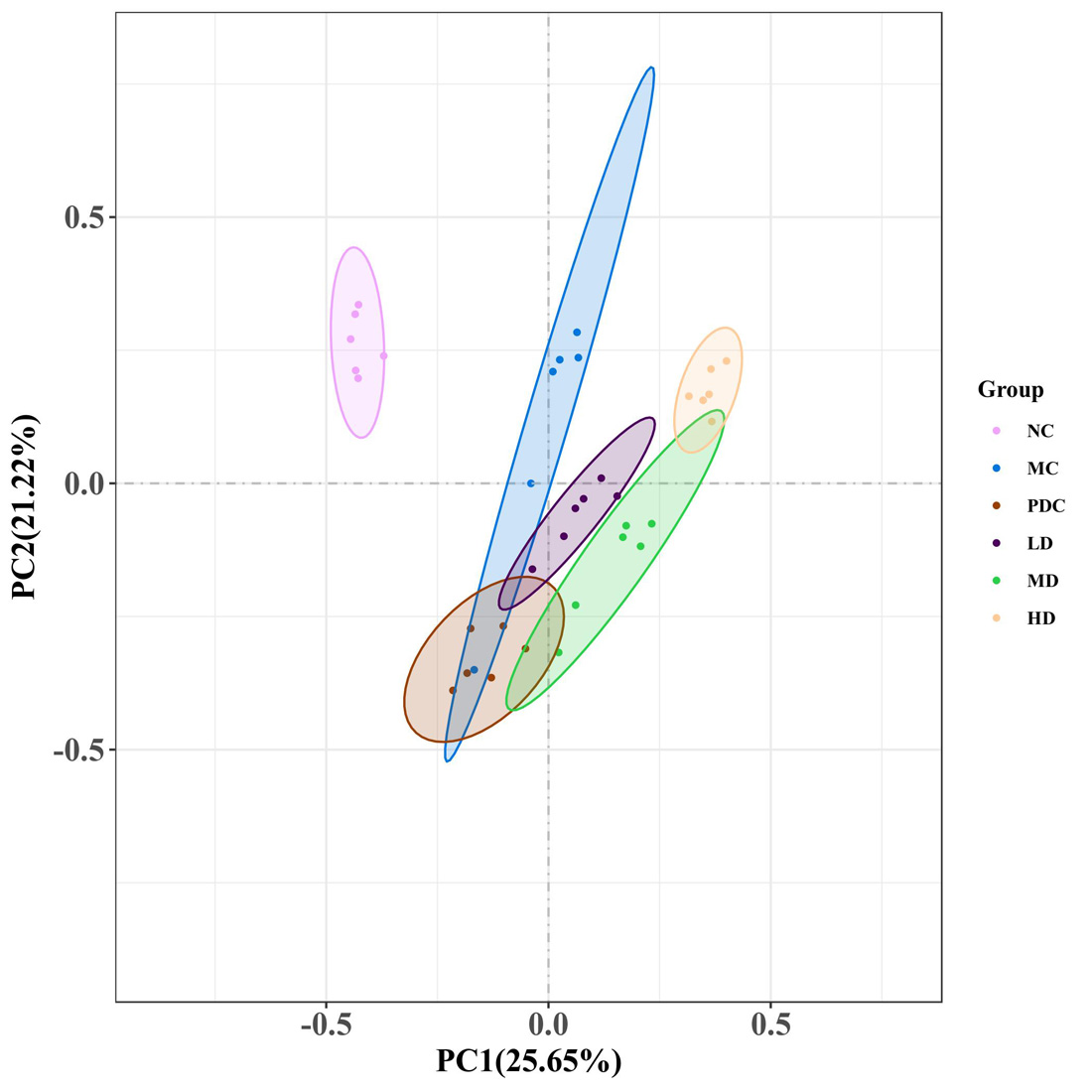

Supplement: Supplementary file 1 [file Data_Sheet_1.zip › Data Sheet 1/Figure 4D.jpg]

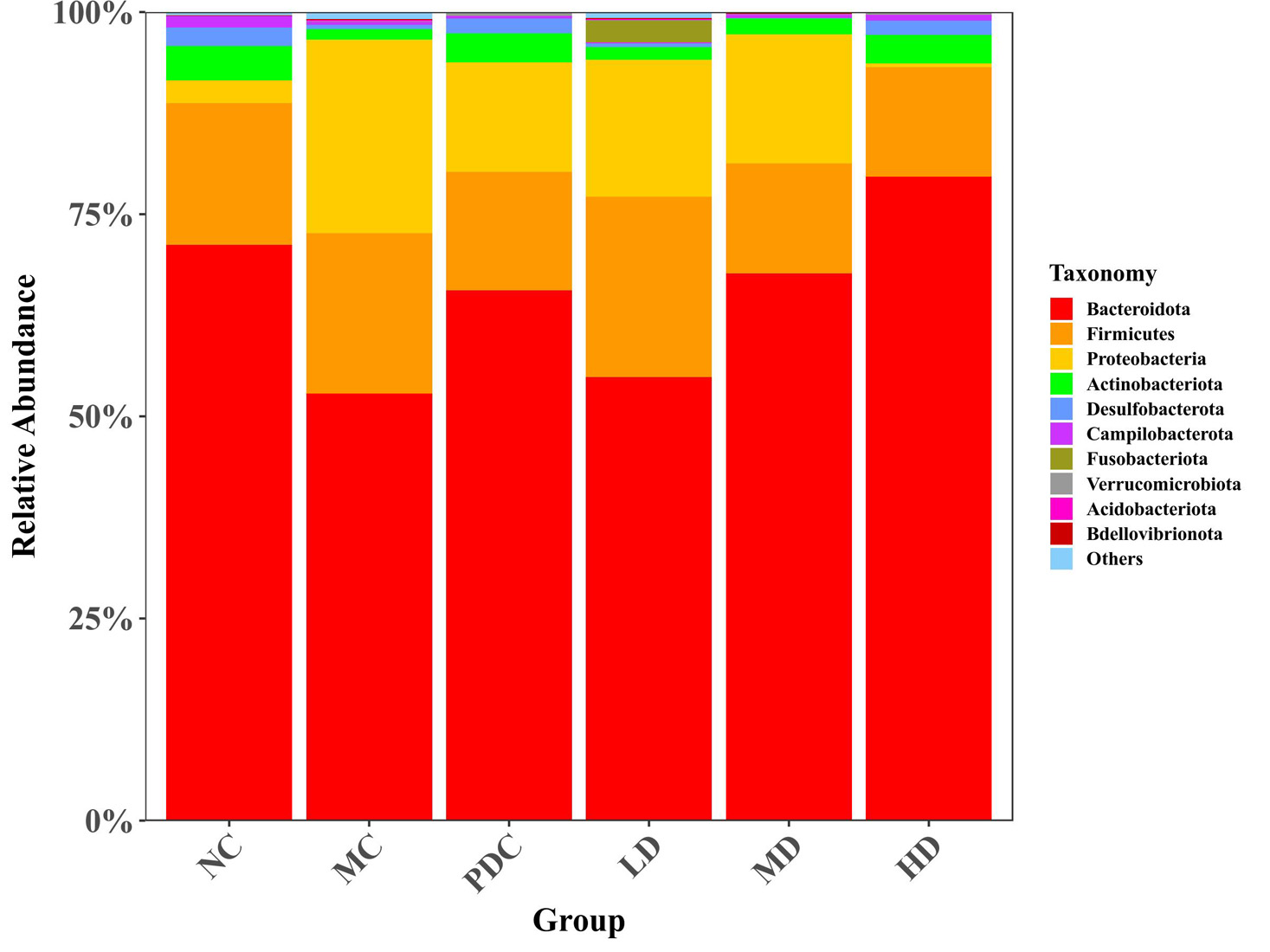

Supplement: Supplementary file 1 [file Data_Sheet_1.zip › Data Sheet 1/Figure 4E.jpg]

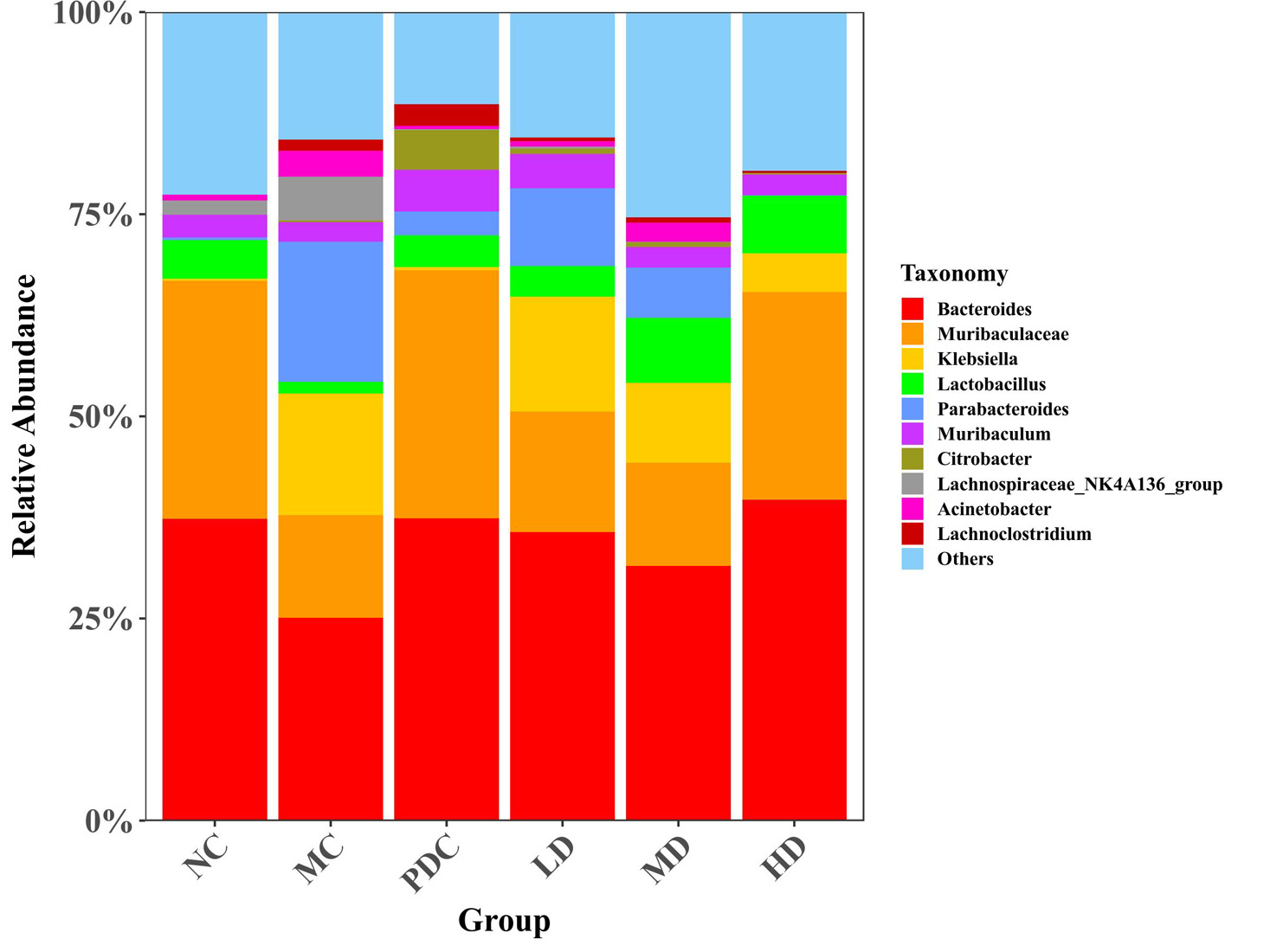

Supplement: Supplementary file 1 [file Data_Sheet_1.zip › Data Sheet 1/Figure 4F.jpg]

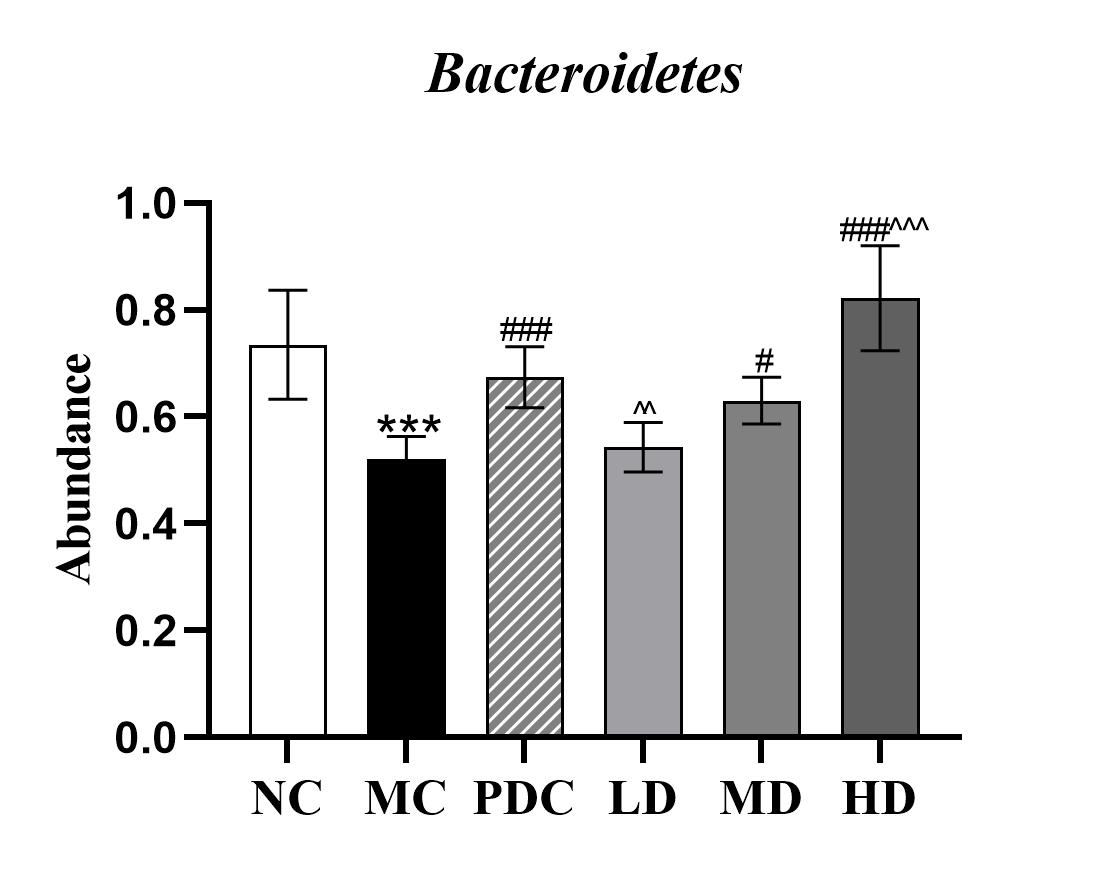

Supplement: Supplementary file 1 [file Data_Sheet_1.zip › Data Sheet 1/Figure 4G.jpg]

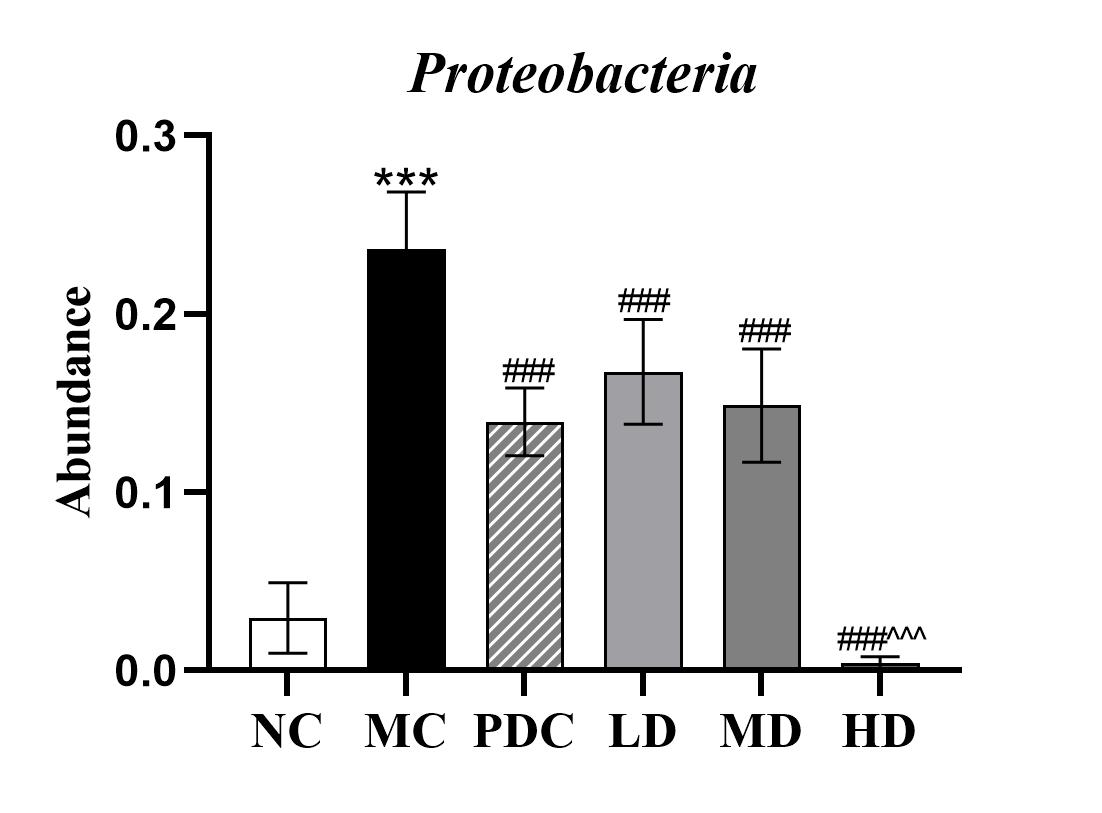

Supplement: Supplementary file 1 [file Data_Sheet_1.zip › Data Sheet 1/Figure 4H.jpg]

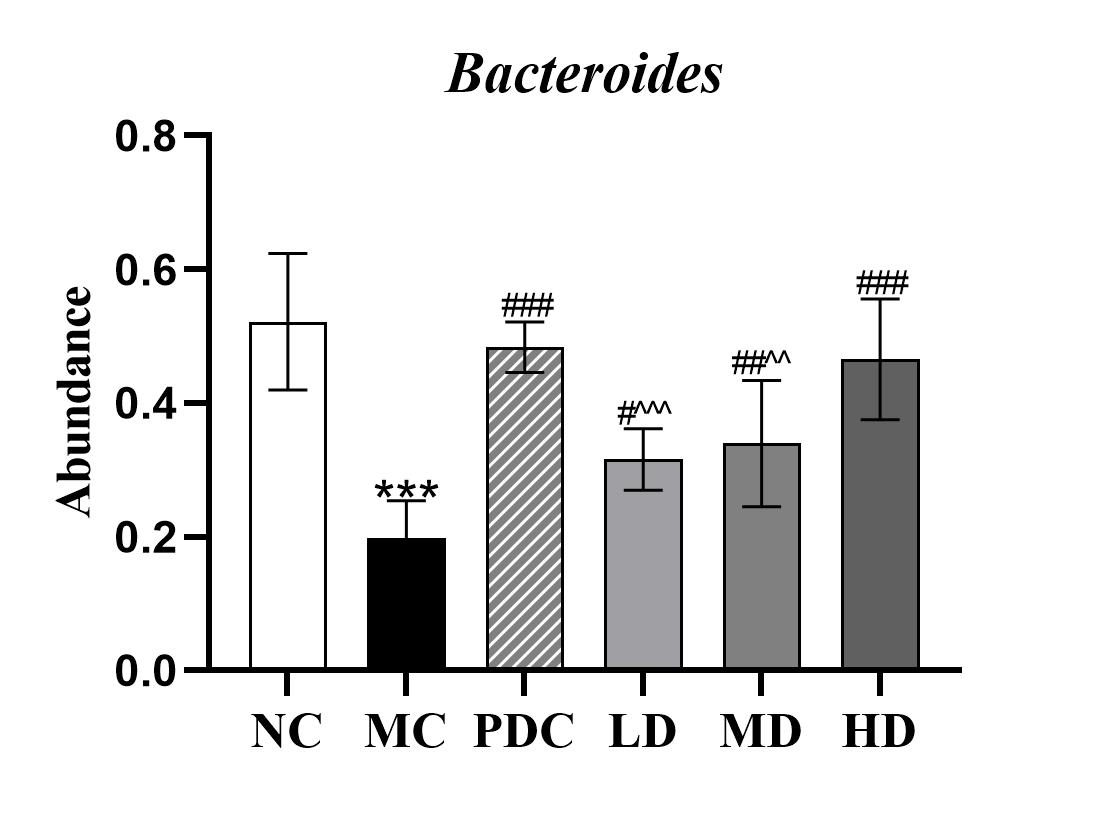

Supplement: Supplementary file 1 [file Data_Sheet_1.zip › Data Sheet 1/Figure 4I.jpg]

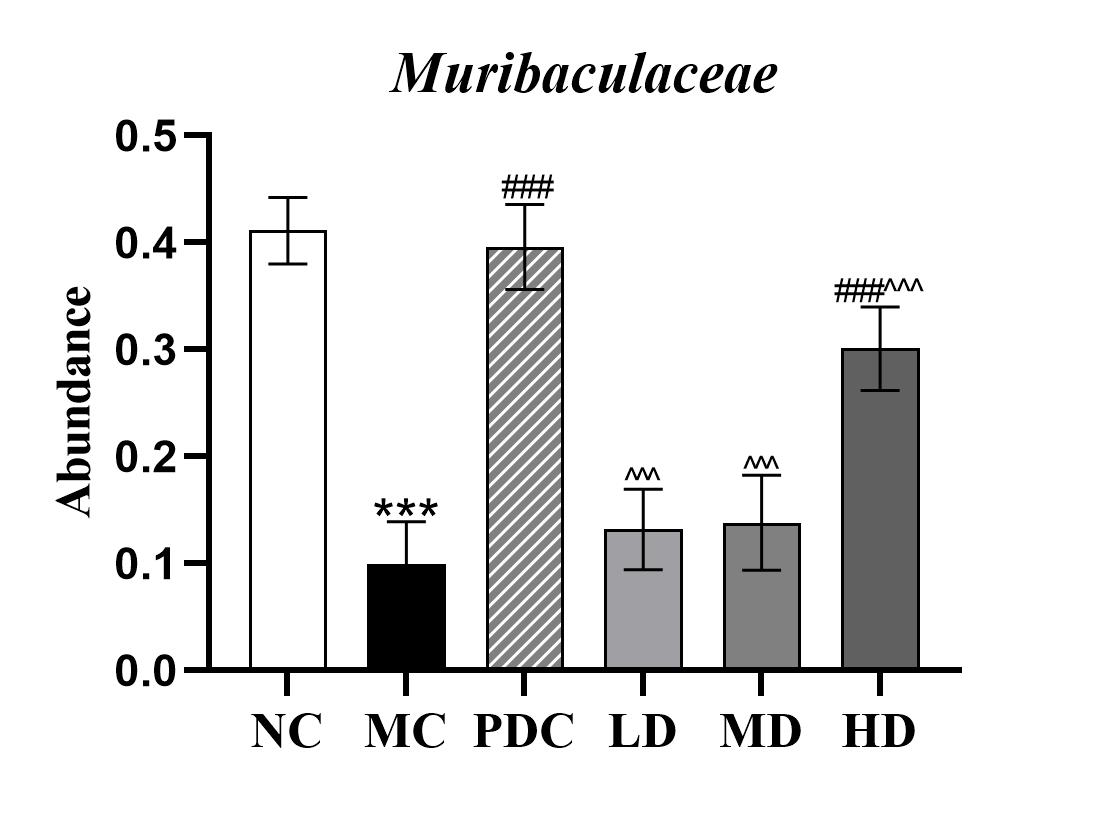

Supplement: Supplementary file 1 [file Data_Sheet_1.zip › Data Sheet 1/Figure 4J.jpg]

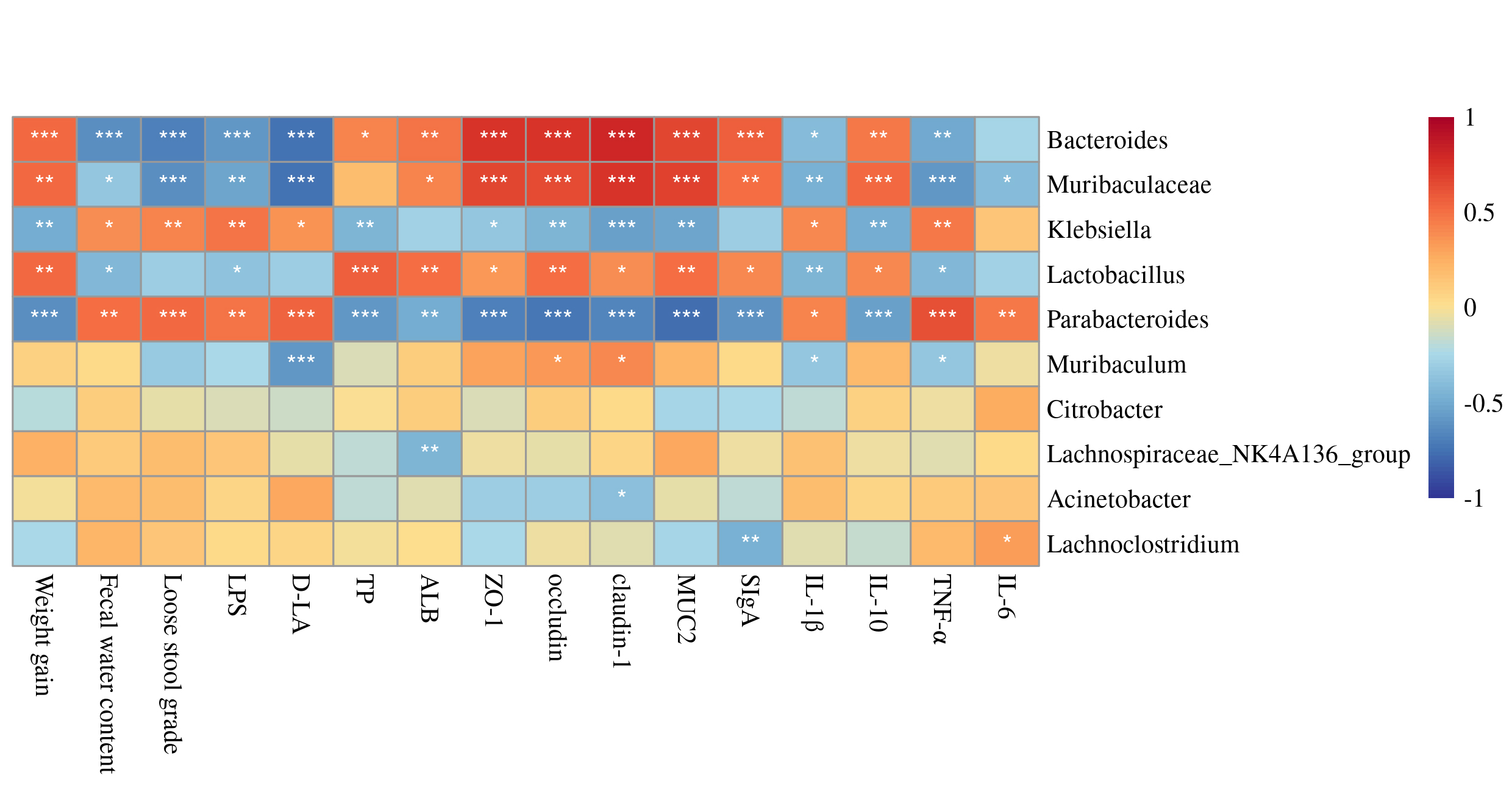

Supplement: Supplementary file 1 [file Data_Sheet_1.zip › Data Sheet 1/Figure 5.jpg]
